# Epigenetic link between Agent Orange exposure and type 2 diabetes in Korean veterans

**DOI:** 10.3389/fendo.2024.1375459

**Published:** 2024-07-12

**Authors:** Sujin Seo, Ye An Kim, Young Lee, Young Jin Kim, Bong-Jo Kim, Jae Hoon An, Heejin Jin, Ah Ra Do, Kyungtaek Park, Sungho Won, Je Hyun Seo

**Affiliations:** ^1^ Department of Public Health Science, Graduate School of Public Health, Seoul National University, Seoul, Republic of Korea; ^2^ Division of Endocrinology, Department of Internal Medicine, Veterans Health Service Medical Center, Seoul, Republic of Korea; ^3^ Veterans Medical Research Institute, Veterans Health Service Medical Center, Seoul, Republic of Korea; ^4^ Division of Genome Science, Department of Precision Medicine, National Institute of Health, Cheongju-si, Republic of Korea; ^5^ Institute of Health and Environment, Seoul National University, Seoul, Republic of Korea; ^6^ Interdisciplinary Program of Bioinformatics, College of National Sciences, Seoul National University, Seoul, Republic of Korea

**Keywords:** Agent Orange, ageing, epigenome-wide association study, microvascular complications, Mendelian randomization, type 2 diabetes

## Abstract

Conflicting findings have been reported regarding the association between Agent Orange (AO) exposure and type 2 diabetes. This study aimed to examine whether AO exposure is associated with the development of type 2 diabetes and to verify the causal relationship between AO exposure and type 2 diabetes by combining DNA methylation with DNA genotype analyses. An epigenome-wide association study and DNA genotype analyses of the blood of AO-exposed and AO-unexposed individuals with type 2 diabetes and that of healthy controls were performed. Methylation quantitative trait locus and Mendelian randomisation analyses were performed to evaluate the causal effect of AO-exposure-identified CpGs on type 2 diabetes. AO-exposed individuals with type 2 diabetes were associated with six hypermethylated CpG sites (cg20075319, cg21757266, cg05203217, cg20102280, cg26081717, and cg21878650) and one hypo-methylated CpG site (cg07553761). Methylation quantitative trait locus analysis showed the methylation levels of some CpG sites (cg20075319, cg20102280, and cg26081717) to be significantly different. Mendelian randomisation analysis showed that CpG sites that were differentially methylated in AO-exposed individuals were causally associated with type 2 diabetes; the reverse causal effect was not significant. These findings reflect the need for further epigenetic studies on the causal relationship between AO exposure and type 2 diabetes.

## 1 Introduction

Type 2 diabetes is a complex metabolic disorder characterised by hyperglycaemia owing to defects in insulin secretion, action, or both (1–4). The pathophysiology in only 10%–15% of type 2 diabetes cases can be attributed to genetic factors, implying the need for research on the environment, lifestyle, and epigenetics of individuals (5). In epigenetic processes, DNA methylation is a crucial regulator of gene expression and molecular phenotypes. Identification of differentially methylated regions (DMRs) associated with exposure to exposome may provide more substantial evidence for causality and may help elucidate associations with complex human diseases (6). Recent epigenetic studies have shown that DNA methylation markers are associated with type 2 diabetes (7–15), and several DMRs associated with *TXNIP*, *ABCG1*, and *SREBF1* have been linked to glycaemic characteristics and diabetic microvascular complications (16, 17).

Agent Orange (AO) is an herbicide that was sprayed across Vietnam and Southeast Asia during the Vietnam War; it contains the most toxic form of dioxin 2,3,7,8-tetrachlorodibenzo-*p*-dioxin (TCDD). Given that TCDD has been documented to possess an epigenetic effect on the body (18–20), it is imperative to take into account its extended half-life in the human body, which ranges from 7 to 11 years (21), and the consequential build-up it may induce. Direct exposure to TCDD can lead to tumorigenesis, including prostate cancer, and development of chronic diseases, including diabetes (22–29). Previous experimental studies had reported that TCDD increased hyperglycaemia via peroxisome proliferator activated receptor-gamma in a rat model of diabetes (24) and that female mice exposed to TCDD during pregnancy had increased susceptibility to diabetes (30). Epidemiological research on the effects of AO exposure indicated a significant association (odds ratio: 2.69, 95% confidence interval: 1.09–6.67) between AO exposure and type 2 diabetes development in Vietnam War soldiers (31). Furthermore, subsequent studies on US veterans have revealed a potential link between AO exposure and type 2 diabetes development (32–34). However, an increase in AO exposure did not increase the incidence of diabetes, according to a meta-analysis of AO exposure (35). Additionally, the US Veterans Affairs Commission concluded that the link and causality between AO exposure and type 2 diabetes development are uncertain (21). However, the studies are limited by the challenges faced when determining the extent of genome-level exposure from actual epidemiological investigations, since a combination of genetic and environmental factors contributes to the development of the disease. The novelty of our study lies in identifying the biochemical mechanism of the association of AO and diabetes.

Recent advances in the interpretation of DNA methylation and bioinformatics have facilitated epigenetic studies on AO exposure (12, 36). Studies have shown that DNA methylation markers are associated with AO exposure and are potential biomarkers of clinical diseases (12, 36). Furthermore, the combination of mediator analysis with genotype and methylation data is expected to help address the concerns regarding the causal relationship between AO exposure and type 2 diabetes development. Therefore, in this study, we aimed to investigate the association factors and causality between AO exposure and type 2 diabetes development in Korean veterans of the Vietnam War. We conducted DNA methylation analysis, which could be divided into the following phases: 1) an epigenome-wide association study (EWAS) of all individuals with type 2 diabetes and of a community-based cohort consisting of a general population without type 2 diabetes to identify type 2 diabetes-related DMPs and 2) an EWAS of Korean veterans with type 2 diabetes (AO-exposed group) and a community cohort consisting of individuals with type 2 diabetes (AO-unexposed controls) to identify AO-related DMPs. Additionally, by combining the genotype and DNA methylation data, an association study was conducted, and causality was assessed using Mendelian randomisation (MR) analysis. Furthermore, using estimated polygenic risk scores (PRSs) for type 2 diabetes, we compared differences in diabetogenic genetic predisposition and epigenetic impact between AO-exposed and AO-unexposed patients with type 2 diabetes.

## 2 Materials and methods

### 2.1 Study design and participants

**Figure 1:**
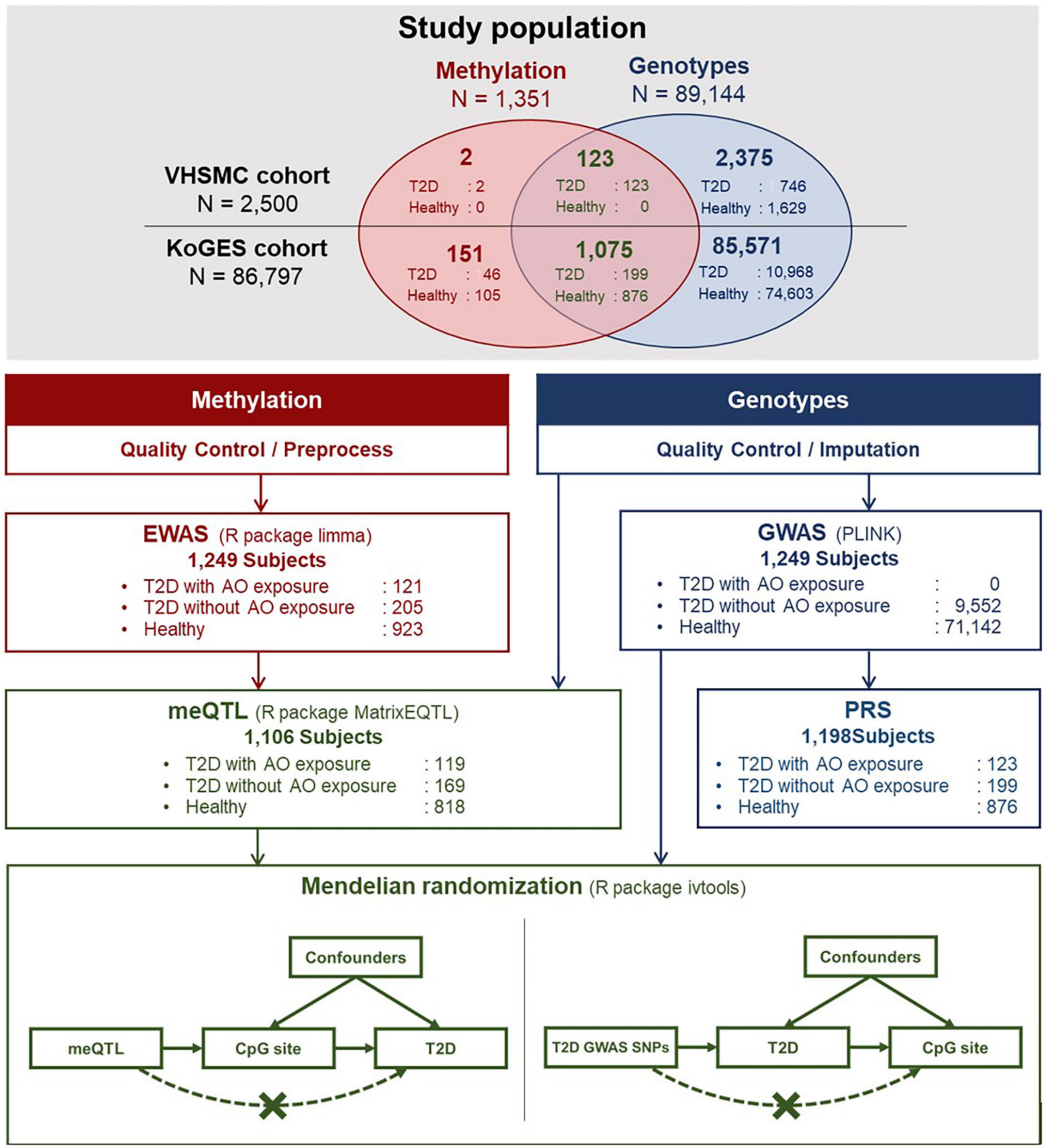
Schematic illustration of the flow of this study. AO, Agent Orange; EWAS, epigenome-wide association study; GWAS, genome-wide association study; KoGES, Korean Genome and Epidemiology Study; meQTL, methylation quantitative trait locus; SNP, single nucleotide polymorphism; T2D, type 2 diabetes; VHSMC, Veterans Health Service Medical Centre.

Clinical and genetic data from the Veterans Health Service Medical Center (VHSMC) cohort (37, 38) and the Korean Genome and Epidemiology Study (KoGES) of the National Biobank of Korea were integrated into this study (sample size, n = 89,297, **Figure 1**) (38, 39). In total, 2,500 and 86,797 individuals from the VHSMC and KoGES cohorts, respectively, were eligible to participate in the study. Adequacy of sample sizes were assessed using *pwrEWAS* (40) considering power and expected effect sizes. The simulation accounted for tissue type (blood samples), number of total and differentially methylated CpGs (80,000, 50), effect size (0.05, 0.1, 0.2), target false discovery rate (0.05), and statistical methods to perform differential methylation analyses(limma). For each parameter set, 50 data sets were simulated and the powers were calculated. AO-exposed participants with type 2 diabetes were selected from the VHSMC cohort based on service-connected disease codes, assigned by official medical examinations for diabetes, with data on their service areas and the duration of their exposure AO exposure during the Vietnam War. Through official medical examinations, the participants were certified as having type 2 diabetes, initially not requiring insulin treatment and responding to oral hypoglycemic agents, along with appropriate test results. The participants with or without type 2 diabetes who were not exposed to AO were selected from the KoGES database and constituted the control group. DNA methylation and genotype analyses involved 125 AO-exposed individuals with type 2 diabetes, 11,959 AO-unexposed individuals with type 2 diabetes, and 77,213 healthy individuals. The exclusion criteria were as follows: 1) individuals who had not passed the pre-process procedure and not fulfilled the quality control (QC) criteria for methylation and 2) individuals who had not passed the QC for genotype and imputation. Finally, 1,249 individuals were included in EWAS, 1,106 individuals were included in the combined analysis of methylation and genotype, and 1,198 individuals were included in PRS modelling.

### 2.2 Ethics

The study protocol was approved by the institutional review board (IRB) of the Veterans Health Service Medical Center (VHSMC) (IRB no. 2018-12-016 and IRB no. 2018-08-032). All participants provided written informed consent before participation. The Korean Genome and Epidemiology Study (KoGES) cohort obtained the informed consent from participants. For control data, the IRB of VHSMC approved the study and waived informed consent from the individuals (IRB no. 2019-06-007), since data analysis was retrospective and the data were de-identified. The study was conducted in compliance with the Helsinki Declaration. The study was conducted with bioresources from the National Biobank of Korea, the Centers for Disease Control and Prevention Agency, Republic of Korea (KBN-2021-042) and (KBN-2020-101).

### 2.3 DNA methylation profiling

Blood samples from the participants were collected in EDTA tubes during baseline visits and corresponding genomic DNA was extracted for methylation profiling. DNA quality was analysed using PicoGreen (Invitrogen, Carlsbad, CA, USA) with the Synergy HTX reader (BioTek, Winooski, VT, USA); DNA purity was assessed using a spectrophotometer (NanoDrop ND 1000UV-vis; Thermo Fisher Scientific, MA, USA) and DNA quality was assessed using gel electrophoresis. High-quality DNA samples (genomic DNA: concentration > 70 ng/μL, volume > 20 μL, total amount > 1.4 μg) were subjected to bisulphite conversion using an EZ DNA methylation kit (Zymo Research, Irvine, CA), according to the manufacturer’s instructions. The DNA methylation fraction values were measured using Illumina Infinium Methylation EPIC BeadChip 850 K (Illumina Inc., San Diego, CA), which can detect 850,000 CpG sites; 90% of the 450,000 sites are in the distal cis-regulatory regions and more than 350,000 CpGs of these are located in enhancer regions. MethylationEPIC BeadChip experiments were performed in accordance with the manufacturer’s instructions, and image BeadChips were constructed in line with the Mac instructions.

The raw signal intensity files were imported, and unreliable intensity values were considered missing values based on the *p*-values, which were calculated by comparing the fluorescence intensity and noise distribution (41). Two colour channels of BeadChips were normalised using the *correct_dye_bias* function from the ewastools package and the data were converted to beta values (the ratio of methylated to unmethylated signal intensities). The pre-processed data were subjected to QC. First, the samples of participants who did not meet the threshold recommended in the BeadArray Control Reporter Software Guide by Illumina were excluded. The sex of each individual was compared with that inferred by the normalised intensities of the CpGs located in the sex chromosomes, and the mismatched individuals were filtered out. Additionally, samples with logarithmic odds of being outliers greater than -4.0 were excluded. Leukocyte compositions were predicted using the function *estimateLC* (42) and data from individuals who had > 30% NK cells or > 9% nRBCs. The estimated cell proportions were not different between groups and are summarised in **Supplementary Table 7**. Individuals with principal component (PC) scores having standard deviations greater than ±1.5 were considered outliers, and hence, were not included in the following analyses. All the described pre-processing steps were performed using the functions from the ewastools package (42) in R. Second, the CpG sites were subjected to QC. We eliminated the CpG sites with a missing rate of ≥ 3%. Single nucleotide polymorphism (SNP)-overlapped probes and cross-reactive probes were removed by referring to the manifest file and provided as a cross-reactive probe list (43). Two cohorts were considered as a batch, and batch effects were adjusted using the ComBat function of the surrogate variable analysis package (44). After QC, 1,249 individuals with 821,509 CpGs remained and were subjected to further analysis. The MDS plot on M-values for the 1,249 individuals was plotted using *plotMDS* with default options from the limma package.

### 2.4 Genotyping and imputation

Genotyping was performed on 89,144 individuals using the Affymetrix 5.0 or 6.0 Array or the Korea Biobank Array (Version 1.0 or 1.1; Affymetrix, Santa Clara, CA, USA) (45). Genotypes were identified by minimising the batch effect using the K-medoid clustering-based method (46). SNPs were removed if the missing rate was > 5%, the *p*-value of the Hardy–Weinberg equilibrium test was < 1 × 10^-5^, or the minor allele frequency was < 0.05. Individuals whose sex information was inconsistent, whose missing rate was > 5%, or whose heterozygosity was < 1 × 10^-5^ were excluded. QC was conducted using ONETOOL (47). Genotype imputation was conducted using the Northeast Asian Reference Database imputation server (https://nard.macrogen.com/) (48), and the SNPs with an INFO > 0.8 were included in the analysis. A total of 81,892 individuals presenting 21,444,246 SNPs were included in subsequent analysis.

### 2.5 Statistical analyses

#### 2.5.1 DMPs associated with AO exposure on disease-risk epigenetic markers

Association between the methylation level of each CpG site and type 2 diabetes development in 121 AO-exposed individuals with type 2 diabetes, 205 AO-unexposed individuals with type 2 diabetes, and 923 healthy individuals was tested with M-values, calculated as the log2 ratio of the methylated probe intensity to the unmethylated probe intensity, using the ‘linear models for microarray data’ (limma) package in R, version 3.6.3 (R Core Team, Vienna, Austria) (49). Age and sex were included as covariates. In addition, we included 10 PC scores to effectively remove the batch effect. An EWAS was conducted to identify DMPs based on the fold-change (FC) and the ratio between cases and controls, using Benjamini–Hochberg correction for controlling the false discovery rate (FDR) (50). The statistical significance of the volcano plot was set at |log_2_FC| > 0.1 and FDR-adjusted *p* < 0.05. The analysis involved two steps, as follows: 1) assessing the relationship between type 2 diabetes development and DNA methylation (type 2 diabetes vs. healthy individuals), which allowed the identification of DMPs related to type 2 diabetes; and 2) assessing the link between AO exposure and type 2 diabetes development using methylation analysis (AO-exposed individuals with type 2 diabetes vs. AO-unexposed individuals with type 2 diabetes). Through the intersection of these two gene sets, we could identify methylation markers in AO-exposed individuals with type 2 diabetes. All individuals with type 2 diabetes, who were exposed to AO, were men, and the same covariates, except for sex, were employed. Differentially methylated regions (DMRs) were also explored with DMRcate. DMRcate function was used with the default options except for the inclusion of a minimum number of 10 CpG sites.

DMPs discovered to be associated with type 2 diabetes were validated with the European Prospective Investigation into Cancer and Nutrition (EPIC)-Norfolk study (7). Methylation intensities were measured using Illumina HumanMethylation450 array in whole blood samples of 1,264 individuals, comprising 563 type 2 diabetes cases and 701 controls. Logistic regression was employed to determine the impact of DMPs on type 2 diabetes after adjusting the effect of age, sex, estimated cell counts, and sample plate. Summary statistics derived from the logistic regression were downloaded from https://www.repository.cam.ac.uk/handle/1810/299058.

Additional sensitivity analysis was performed with a more homogenous population. All female participants were found to be healthy, with none identified as having type 2 diabetes. To eliminate the potential bias caused by this imbalance, we conducted EWAS exclusively with male participants and compared their estimates with the results obtained from all the study participants. Ages were significantly different between cases and controls (*p* < 0.0001, **Table 1**), and to address potential confounding by age, we performed the same process with only sensitivity analyses to assess older participants, aged above the median age of all participants. Further, since our analyses included individuals with an average age of 60 years, including those with a history of cancer, this could potentially result in inaccurate conclusions. Therefore, we conducted an additional analysis, excluding patients with a history of cancer. Moreover, we conducted subgroup analyses on type 2 diabetes microvascular complications, including diabetes mellitus (DM) CKD and DM retinopathy. Patients were categorised based on the type of complication. We assumed those with unknown DM microvascular complication status in the AO-unexposed group to have no complication. Next, we conducted separate subgroup analyses for patients with each complication and compared the coefficients of AO for the methylation level to those obtained from EWAS results using all patients with type 2 diabetes.

**Table 1:** Baseline characteristics of the study population of the EWAS study.

	Entire	VHSMC cohort	KoGES cohort (AO-unexposed)			p
		AO-exposed type 2 diabetes	AO-unexposed type 2 diabetes	Healthy control (non-DM)		
				Male	Female	
	(N = 1,249)	(N = 121)	(N = 205)	(N = 505)	(N = 418)	
Age (years), median (IQR)	58 (52, 69)	73 (71, 74)	62 (55, 69)	57 (52, 66)	54 (51, 63)	<0.0001^a^ (A, B, C)
BMI (kg/m^2^), mean ± sd	24.1 ± 2.9	25.21± 3.0	25.0 ± 2.8	23.8 ± 2.8	23.8 ± 2.9	<0.0001^a^ (B, C)
Duration of type 2 diabetes (years), median (IQR)	15.4 (7.2, 23.8)	25.0 (20.0, 31.0)	8.1 (4.7, 15.1)	–	–	<0.0001^b^
Onset age of type 2 diabetes (years), median (IQR)	50.0 (43.2, 55.8)	48.0 (41.0, 54.0)	50.9 (45.1, 57.6)	–	–	0.0015^b^
HbA1c (%), mean ± sd	5.9 ± 1.0	7.4 ± 1.4	7.2 ± 1.0	5.5 ± 0.4	5.4 ± 0.2	<0.0001^a^ (A, B, C)
eGFR (mL/min/1.73 m^2^), median (IQR)	72.7 (63.5, 79.6)	53.0 (42.0, 63.0)	73.2 (64.1, 81.8)	75.1 (69.1, 82.6)	71.9 (66.0, 76.9)	–
Creatinine (mg/dL), mean ± SD	1.0 ± 0.3	1.5 ± 0.7	1.1 ± 0.4	1.0 ± 0.1	0.8 ± 0.1	<0.0001^a^ (A, B, C)
Comorbidity						
Ischaemic heart disease, n (%)	38 (3.0)	35 (28.9)	1 (0.5)	2 (0.4)	0	<0.0001^c^
Hypertension, n (%)	195 (15.6)	102 (84.3)	27 (13.2)	41 (8.1)	25 (6.0)	<0.0001^d^
DM CKD (60), n (%)	173 (13.9)	81 (66.9)	34 (16.6)	31 (6.1)	27 (6.5)	<0.0001^d^
DM retinopathy, n (%)	56 (4.5)	56 (46.3)	–	–	–	–
Cancer, n (%)	24 (1.9)	24 (19.8)	0	0	0	<0.0001^c^
Prostate cancer	9 (0.7)	9 (7.4)	0			–
Lymphoma	2 (0.2)	2 (1.7)	–	–	–	–
Colon cancer	3 (0.2)	3 (2.5)	–	–	–	–
Gastric cancer	4 (0.3)	4 (3.3)	0	0	0	–
Pancreatic cancer	2 (0.2)	2 (1.7)	0	0	0	–
Leukaemia	1 (0.1)	1 (0.8)	–	–	–	–
Liver cancer	3 (0.2)	3 (2.5)	0	0	0	–
Other	1 (0.1)	1 (0.8)	–	–	–	–
Stroke, n (%)	35 (2.8)	27 (22.3)	5 (2.4)	1 (0.2)	2 (0.5)	<0.0001^c^

VHSMC cohorts (Veterans Health Service Medical Center cohort): AO-exposed type 2 diabetes.

KoGES cohort (Korean Genome and Epidemiology Study): AO-unexposed individuals with type 2 diabetes and healthy individuals.glycated haemoglobin (HbA1c), estimated glomerular filtration rate (eGFR), chronic kidney disease (CKD).

a ANOVA and Tukey’s Honest test results in parentheses at 0.05 significance level after multiple comparison adjustment (A: AO-exposed T2D vs. AO-unexposed T2D, B: Healthy vs. AO-exposed T2D, C: Healthy s AO-unexposed T2D).

b Yuen trimmed mean t-test.

c Fisher’s exact test.

d Chi-square test.

#### 2.5.2 Methylation quantitative trait locus analysis and MR analysis

Methylation quantitative trait locus (meQTL) analysis was performed; the SNPs within 500 kb (*cis*) of the identified DMPs were pruned to consider the linkage dependency of SNPs in further analysis. The linear model for each methylation–SNP pair after adjusting for age and sex was tested using the MatrixEQTL package at an FDR-adjusted significance level of 0.05 (51).

To investigate whether the identified DMPs play a causal role in type 2 diabetes or whether type 2 diabetes affects the methylation levels, we performed a one-sample MR analysis using the methylation and genotype data of 1,106 individuals, including 119 AO-exposed individuals with type 2 diabetes, 169 AO-unexposed individuals with type 2 diabetes, and 818 healthy individuals (**Figure 1**). The pairs of SNPs and methylation levels that were significantly associated but were not associated with type 2 diabetes were utilised in the MR analysis using the two-stage estimation method. We assumed that the methylation level of the CpG sites detected in DMPs affects type 2 diabetes predisposition, and SNPs were used as instrumental variables. First-stage regression estimated the regression coefficient of an SNP at the methylation level. Next, the predicted methylation level was plugged into the second-stage logistic regression to estimate the effect of methylation level on type 2 diabetes. Further analysis to check the existence of a reverse causation effect was conducted using a two-stage estimation method. The SNPs with genome-wide significant associations were used to confirm whether the instrumental variable was associated with type 2 diabetes development. The estimated bias of the binary model was adjusted using the control function, as suggested by Vansteelandt, with the ivtools package (52).

#### 2.5.3 Estimation of PRSs for type 2 diabetes development

A genome-wide association study (GWAS) of 80,694 individuals from the KoGES cohort was performed (**Figure 1**), and summary statistics were used to calculate the PRS. For replication analysis, summary statistics from GWAS and BioBank Japan (BBJ) were used (https://pheweb.jp/) (53). Further details on PRS analysis and pathway PRS can be found in **Supplementary Data Sheet 2**. All the codes for analysis in this study can be downloaded from https://github.com/wonlab-healthstat/Methylation_AO_T2D.

## 3 Results

### 3.1 Baseline characteristics

The baseline characteristics of the study participants are presented in **Table 1**. All individuals with type 2 diabetes were men, whereas 43% of the healthy individuals were women. The age of the AO-exposed individuals with type 2 diabetes was higher than that of the AO-unexposed individuals and that of healthy individuals (*p* < 0.0001). The duration of type 2 diabetes in AO-exposed individuals with type 2 diabetes was longer than that in AO-unexposed individuals (26.22 ± 8.48 vs. 10.85 ± 8.39 years, *p* < 0.0001). The onset age of type 2 diabetes was lower in AO-exposed individuals with type 2 diabetes than in AO-unexposed individuals (46.78 ± 8.46 vs. 50.84 ± 9.37 years, *p* < 0.0001). The level of glycated haemoglobin (HbA1c) in AO-exposed individuals with type 2 diabetes differed from that in AO-unexposed individuals with type 2 diabetes (7.40% ± 1.34% vs. 7.22% ± 0.98%, *p* = 0.0359). The estimated glomerular filtration rate was the lowest in AO-exposed individuals with type 2 diabetes (*p* < 0.0001). The incidence of comorbidities in AO-exposed individuals with type 2 diabetes was higher than that in AO-unexposed individuals (*p* < 0.0001) and all the patients with cancer history were in AO-exposed type 2 diabetes.

### 3.2 Association between AO exposure and DNA methylation

After performing the QC procedures, DMPs of 1,249 individuals with 821,509 CpG sites, including 121 AO-exposed individuals and 1,128 controls (205 AO-unexposed individuals with type 2 diabetes and 923 healthy individuals), were analysed (**Supplementary Figure 1**). The power analyses show that to detect effect sizes as small as 0.05 with 80% power, 250 subjects are required (**Supplementary Figure 7**), which supports that the number of sample sizes is sufficient. The MDS plot did not reveal any distinct cluster, and Q-Q plots indicated no inflation of analysis with the inflation factors of 1.1 and 1.04 for type 2 diabetes vs. healthy and AO-exposed type 2 diabetes vs. AO-unexposed type 2 diabetes, respectively, thereby indicating the absence of population genetic structure. The volcano plot is shown in **Supplementary Figure 2**.

**Figure 2:**
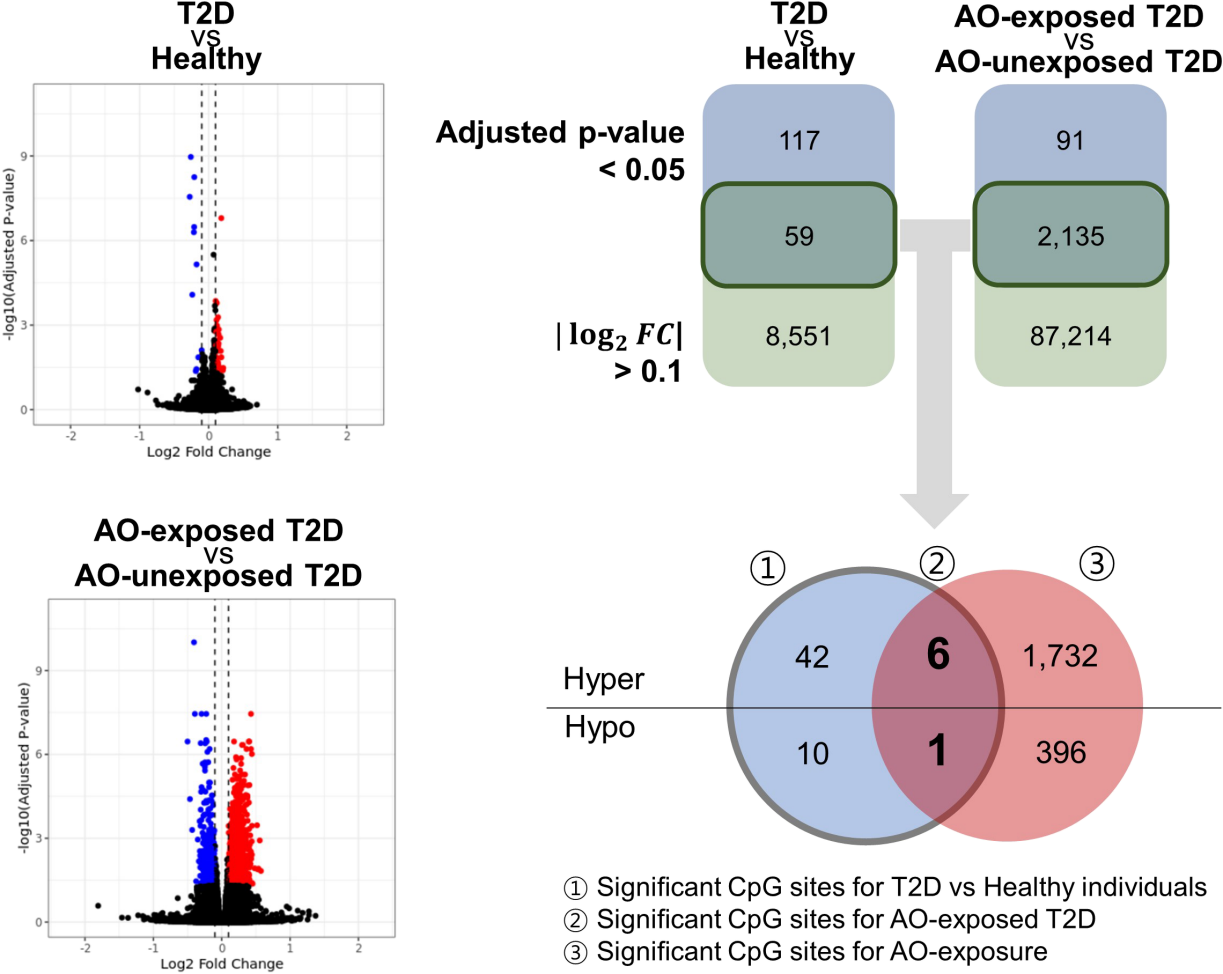
Volcano plot and Venn diagram showing the significant CpG sites of all the groups studied. The number of significant CpG sites at a significance level of 0.05, with an absolute log_2_(fold-change) > 0.1. Significant CpG sites that met the two criteria were further categorised as hyper/hypo. DM, diabetes mellitus; AO, Agent Orange; T2D, type 2 diabetes; hyper, hypermethylation; hypo, hypomethylation.

**Figure 3:**
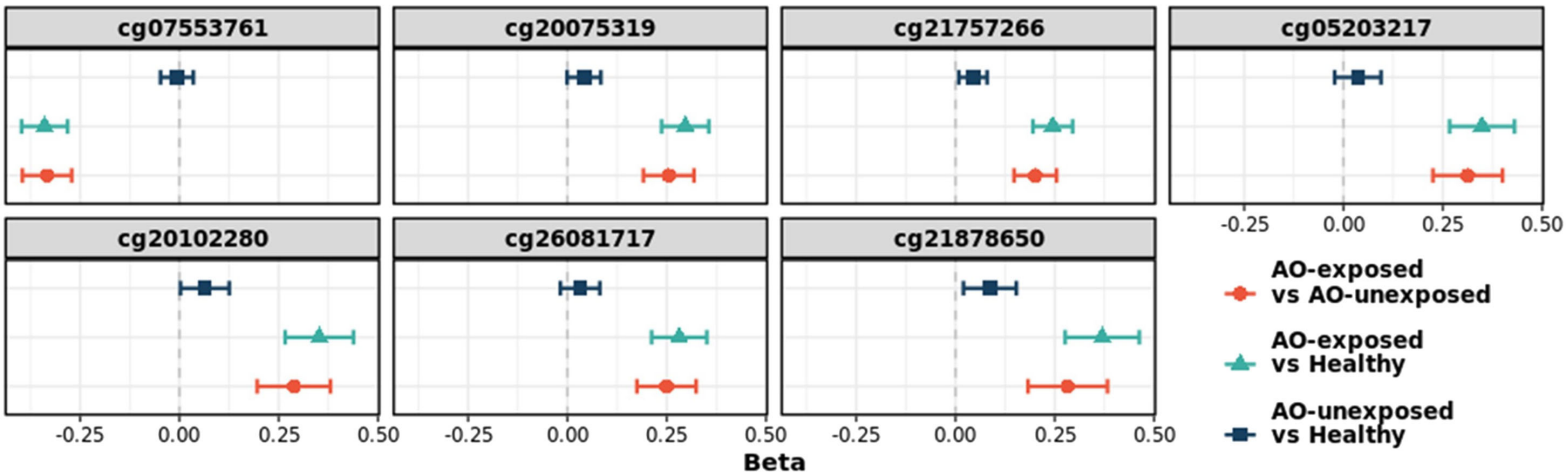
Change in the log_2_(fold-change) values of seven CpG sites. The sites were identified as significant in type 2 diabetes versus healthy individuals, and in AO-exposed individuals with type 2 diabetes versus AO-unexposed individuals. AO, Agent Orange; Chr, chromosome; T2D, type 2 diabetes; FC, fold-change.

Fifty-nine CpGs were significantly associated with the DM phenotype (AO-unexposed individuals with type 2 diabetes and AO-exposed individuals with type 2 diabetes) compared to those of healthy individuals (**Figure 2**). Furthermore, the DMPs between AO-exposed individuals with type 2 diabetes and AO-unexposed individuals revealed that 2,135 CpG sites were significantly associated with AO exposure (**Figure 2**). The Venn diagram shows that seven CpG sites were significantly associated with both DM phenotype and AO-exposed individuals with type 2 diabetes (**Figure 2**). They consisted of six hyper-methylated CpG sites (cg20075319, cg21757266, cg05203217, cg20102280, cg26081717, and cg21878650, which are associated with *KCND3*, *FLT1*, *KCNS1*, *HTR2A*, *TMEM246*, and *ADAMTS6*, respectively) and one hypo-methylated CpG site (cg07553761, which is associated with *TRIM59*). The highest methylation level was observed in AO-exposed individuals with type 2 diabetes, followed by AO-unexposed individuals and healthy individuals for the hyper-methylated CpGs, and vice versa for the hypo-methylated CpG, when comparing the absolute log_2_FC values (**Figure 3**, **Supplementary Table 2** **).** DMR analysis did not reveal any significant association. Validation was carried out using participants from the EPIC-Norfolk study. Out of 59 significant CpG sites, analysis results were available for 18 CpG sites. Directions of the estimates were consistent for 14 CpG sites, and among them, 6 CpGs were significant at the significance level of 0.05 (**Supplementary Table 3**). Sensitivity analyses, to assess the robustness of our analysis, showed the consistent direction of all estimates and similar effect sizes (**Supplementary Figure 3**). Subgroup analyses for each DM microvascular complication resulted in similar estimates. Estimates for the seven identified CpGs are listed in **Supplementary Table 4**.

### 3.3 meQTL analysis and MR analysis

To assess whether the CpGs associated with AO have a causal effect on type 2 diabetes, we considered the *cis-*SNPs within 500 kb from the CpGs, and meQTL was found by testing the correlations for 2,569 SNP-CpG pairs in 59 CpG sites. Significant correlations were found for 134 pairs at an FDR-adjusted significance level of 0.05 (**Supplementary Figures 4, 5**, **Supplementary Table 5**). They were utilised for MR, and a causal effect was found for six CpGs, namely cg06827192, cg20075319, cg20102280, cg22400605, cg26081717, and cg26826927, which are associated with *CNKSR3*, *KCND3*, *HTR2A*, *LOC101929532*, *TMEM246*, and *COG5*, respectively (**Table 2**, **Supplementary Table 8**). Among these, cg20075319, cg20102280, and cg2608171 were significantly associated with AO exposure, according to the DMP analysis. Their log_2_FC values (adjusted *p*-values) were 0.2555 (9.40 × 10^-11^), 0.288 (1.78 × 10^-6^), and 0.250 (1.87 × 10^-7^), respectively. Conversely, to investigate the influence of type 2 diabetes on the CpG methylation levels, GWAS SNPs were used as instrumental variables, and none of the CpGs showed reverse causality (**Figure 1**, **Supplementary Table 6**). These findings implied that alterations in DMPs in relation to diabetes were not caused by type 2 diabetes, but rather by AO exposure.

**Table 2:** Mendelian randomisation analysis of significant associations between CpGs (mediators) and type 2 diabetes (outcomes).

CpG	Chr	Position	Associated gene	Location in gene	Location in CpG island	F^a^	Estimate	Standard error	*p*	Adjusted *p*
cg20075319	1	112332718	*KCND3*	body	Open sea	31.7	-2.5262	0.4707	7.99E-08	1.02E-05^††^
cg26826927	7	107103667	*COG5*	body	Open sea	112.9	-2.0690	0.5318	1.00E-04	0.0127^†^
cg20102280	13	47470793	*HTR2A*	body	Open sea	15.4	-1.3341	0.3491	1.33E-04	0.0166^†^
cg26081717	9	104249747	*TMEM246*	TSS1500	Shore	20.6	-1.3761	0.3705	2.04E-04	0.0230^†^
cg06827192	6	154730156	*CNKSR3*	body	Open sea	36.0	-1.0452	0.2895	3.06E-04	0.0325^†^
cg22400605	2	162975451	*LOC101929532*	body	Open sea	30.6	-1.4692	0.4177	4.36E-04	0.0454^†^

^†^0.01< Benjamini–Hochberg adjusted *p* < 0.05.

^††^Benjamini–Hochberg adjusted *p* < 0.01.

a Significant CpG sites from Mendelian randomisation analysis. The SNPs with the highest correlation with each CpG are listed and their F-statistics are listed as a measure of the instrument’s strength.

### 3.4 Estimation of the PRSs for type 2 diabetes

The PRS of individuals in both type 2 diabetes groups (AO-exposed and AO-unexposed individuals with type 2 diabetes) was higher than that of healthy individuals (*p* < 0.05, **Supplementary Figure 6**). However, no significant difference in PRS values was found between the AO-exposed individuals with type 2 diabetes and the AO-unexposed individuals (*p* > 0.05, **Supplementary Figure 6**, **Supplementary Data Sheet 2**). Further, the relationships between AO exposure and specific pathogenic pathways in type 2 diabetes were investigated by calculating the pathway-specific PRS. Pathways related to adipocytokine signalling, apoptosis, insulin signalling, and maturity onset diabetes of the young were examined. However, through the comparisons between AO-unexposed and AO-exposed groups, no statistically significant differences were found in pathway enrichment, indicating a lack of discernible pathway-level variations associated with AO exposure in the context of type 2 diabetes pathogenesis.

## 4 Discussion

Our investigation revealed that seven DMPs were associated with both AO exposure and type 2 diabetes, and MR analysis for causality demonstrated that AO exposure was a causal epigenetic factor of type 2 diabetes in Korean veterans who were involved in the Vietnam War. The PRS was different between the individuals in both type 2 diabetes groups and healthy individuals; however, there was no change with or without AO exposure, indicating that AO exposure can cause type 2 diabetes development via epigenetic modifications in individuals with a genetic predisposition to type 2 diabetes. Additionally, the 2,135 AO-related DMPs identified in our study may be associated with conditions such as cancer, post-traumatic stress disorders, and Parkinson’s disease, which have been linked to AO exposure in epidemiological studies (22, 31, 34); therefore, additional research in this direction is recommended.

The relationship between AO exposure and type 2 diabetes development had previously been investigated by performing an epidemiological study involving Korean veterans and the Air Force Health Study cohort (31–33). Subsequent investigations based on this cohort demonstrated that AO exposure is associated with type 2 diabetes development (34). In addition to these epidemiological investigations, data from cellular and animal studies supported the potential link between AO exposure and type 2 diabetes development (54). Previous research has revealed that mice lacking the aryl hydrocarbon receptor have increased insulin sensitivity and glucose tolerance, indicating that the receptor plays a physiological role in glucose metabolism (54). A study employing a transcriptome approach to investigate the putative molecular pathways of AO-induced alterations in pancreatic islet and cell insulin secretion (55) identified the potential mechanisms underlying the metabolism-modulating effects of AO, which are essential for the progression of metabolic diseases. However, a meta-analysis using a random-effects model showed that increased AO exposure is not associated with an increased risk of type 2 diabetes (35). Despite this negative finding, dose–response relationships between the dioxin level and type 2 diabetes incidence were observed in several other analyses that controlled the confounding variables (35). Such contradictory findings need to be interpreted considering the peculiarities of epidemiological studies, and epigenomic analysis is expected to provide a more sensitive interpretation of the results (36).

Recent studies on the effect of AO toxicity, using DNA methylation analysis, demonstrated a significant association of dioxin exposure with DNA methylation in *IGF2* and *CYP1A1*, which are potential biomarkers for dioxin exposure in the Vietnamese population (56). In addition, previous studies on DNA methylation had identified four CpG sites located on *TEAD3* in the sperm (36), and DNA methylation for *SLC9A3* in adipose tissue and *PTPRN2* and *SMO* in whole blood has been reported (57) in veterans exposed to AO in Operation Ranch Hand. Here, we investigated the association of DNA methylation with AO-exposed type 2 diabetes and found three significant CpG markers with DMP and MR analyses. Following are the type 2 diabetes junctions in genes, corresponding to the seven DMPs that were supportive of the data obtained here. *KCND3* and *KCNS1*, which regulate the potassium channel function, expressed at low levels in the pancreas (58). Recent research analysing the transcriptome signature in adult human islets has revealed a significant increase in *KCND3* mRNA specifically in alpha cells, along with glucagon (59). RNA sequencing analysis revealed that *KCND3* was the only upregulated gene and a significant biomarker in diabetic kidney disease (60). *FLT1*, also known as VEGFR-1, serves as a receptor for VEGFA in the pancreas (61). *FLT1* knockout mice showed disorganized islet vascularity and impaired insulin secretion (62). *HTR2A*, which regulates the level of serotonin receptor 2A are present in islets of mice and humans (63, 64). 5-HT2A receptors regulate insulin secretion in human islets; a 5-HT2A receptor agonist evoked a 1.5-fold increase in glucose-induced insulin secretion (GSIS), while a 5-HT2A antagonist inhibited GSIS in human islets (64). In a GDM study, *TMEM246* showed abnormally methylated results (65). *ADAMTS6* encodes a secreted metalloprotease, and showed the alleviating effect of insulin on diminishing ADAMTS6 levels in human cell lines, suggesting a weak link with diabetes (66). *TRIM59*, a negative regulator of kappaB kinase/NF-kappaB signalling (67), is associated with reduced GSIS and pancreatic β cell failure in animal models (68, 69). Among the genes, *KCND3*, *FLT1*, *HTR2A*, and *TRIM59* demonstrate associations with beta cell dysfunction and GSIS. While there is also evidence linking *TMEM246* and *ADAMTS6* to diabetes, the association is ambiguous due to a lack of *in vivo* or human cell experiments. Despite the limitations, it is speculated that these genes may be related to beta cell function. Further, the genes associated with significant CpGs for T2D, *CNKSR3*, *COG5*, and *LOC101929532*, have the potential molecular mechanisms underlying T2D. *CNKSR3*, a gene highly expressed in renal collecting ducts, regulates sodium transport and is upregulated by aldosterone, is targeted in protecting kidney disease progression in T2D (70) representing a candidate gene for further investigation in the context of T2D pathogenesis. Additionally, *COG5*, which participates in Golgi vesicle tethering and intra-Golgi transport, hint at intricate cellular mechanisms that may indirectly influence insulin secretion and glucose homeostasis (71). While *LOC101929532* may not have been extensively characterized in the literature, its association in our study underscores its potential relevance to T2D pathology, warranting further investigation. However, MR analysis revealed that AO exposure was a causal factor for type 2 diabetes, whereas reverse MR revealed that the type 2 diabetes-related SNPs did not influence the DMPs identified in this study. These findings supported the causal relationship between AO exposure and type 2 diabetes development. In our study, the duration of type 2 diabetes in AO-exposed individuals with type 2 diabetes was longer than that in AO-unexposed individuals. There may be a connection between AO exposure and the development of young-onset DM, and this could be related to insulin deficiency versus insulin resistance in terms of the mechanism of diabetes. Several studies suggest that methylation in the *KCNQ1* locus is related to insulin sensitivity (72, 73). In addition, epigenetic age measurements with DMR change could potentially be used as a biomarker associated with type 2 diabetes development (74, 75). Since the age and diabetes occurrence mechanisms are related to diabetic complications, they are currently being actively researched, and further research is still recommended. Since methylation is a complex issue related to ageing, insulin sensitivity, and diabetes complications, we compared the PRS constructed from genotype data and found non-significant differences between AO-exposed and AO-unexposed individuals with type 2 diabetes. Owing to the polygenic nature of diabetes, upon AO exposure on the battlefield, individuals with a genetic predisposition for type 2 diabetes may undergo epigenetic modifications related to type 2 diabetes development. A major strength of our study was that it provided epigenetic evidence of molecular determinants of the susceptibility of AO-exposed individuals to develop type 2 diabetes. In addition, type 2 diabetes and significant DMPs were identified by combining the genotype and DNA methylation data with causality analysis. Our study possesses a distinctive advantage as it scrutinized the precise mechanisms underlying the progression of AO and diabetes. Furthermore, it delves into unexplored information that was previously unknown in prior research. In addition, the analysis considered the fact that type 2 diabetes is associated with methylation, and an effort was made to avoid false positives using a strategy that yielded results more accurate than those obtained from the analysis of DMPs associated with multiple AO exposure. However, the study has several limitations. The first is the long time-lag between AO exposure and the methylation level analysis using the Infinium 850 K Bead Chip. Since the half-life of AO is 7–11 years (21), AO would have been degraded to an undetectable level and would have had an epigenetic effect on the body during that duration. In addition, the individuals were exposed to high doses of AO employed for military use, whereas a few control individuals may have been occupationally exposed (dioxin-industry workers, among others). In this study, a sufficient number of control individuals were enrolled to reduce the above-mentioned possibility, which was predicted to have reduced the frequency of false-positive dioxin exposure-related methylation results. It can be assumed that Korea veterans will have a similar median half-life of 2,3,7,8-TCDD to that of Ranch Hand veterans, whose serum samples taken in 1982 and 1987 revealed a median half-life of 7.1 years (95% confidence interval for median 5.8-9.6 years) (76). Second, the average age of AO-exposed group individuals was higher than that of the control group individuals. According to the epidemiological distribution of the population, there was an age bias in the cohort of individuals who participated in the Vietnam War, although that was not the case in the control group. Considering that different ages might influence the methylation level, all data sets were analysed by adjusting the age of the participants. Although genotype is largely independent of age, it was still included in the analysis with PRS. Third, in our study, among 125 AO-exposed type 2 diabetes veterans, we targeted those who were recognised as exposure group of AO for Korean Veterans (https://vadisabilitygroup.com/agent-orange-exposure-benefits-for-korean-veterans/) and this was a special group receiving state financial support. The remaining 2,500 patients participated in a chronic disease cohort study conducted by Veterans Hospital. Researchers will be interested in determining the prevalence of individuals with AO-exposed type 2 diabetes among the global diabetic population. Based on the global population of type 2 diabetes patients being 529 million (77), it is approximately 0.4% for the estimated percentage of type 2 diabetes patients worldwide who have developed type 2 diabetes associated to AO exposure (2 million in Vietnamese and American veterans, 73,647 in South Korea). Fourth, women participated in the Vietnam War; however, they were non-combatants, and only a few of them were exposed to AO. Therefore, the AO exposure group was exclusively composed of men. Thus, this can result in sex bias, since some specific patterns of DNA methylation may exist in women. Even though, sex variable was controlled in the model, our results still can be biased. Further studies are necessary. There could be more unmeasured confounders for methylation. The authors attempted to assess population stratification through MDS plots; however, unlike genetic analysis, where Mendelian laws govern and there are relatively few confounders, methylation can be influenced by environmental factors and, therefore, have more potential confounders. As MR is robust against uncontrolled confounders, it was applied to assess the significance of CpGs in our study. In addition, despite the authors’ inclusion of overlapping data from the comparison of AO with non-AO and the comparison of non-AO type 2 diabetes with healthy individuals, it cannot be asserted that the observed overlapping signals are directly attributable to or associated with AO exposure. There may be additional characteristics, such as smoking status, sweet beverage consumption, and exercise frequency, which should be considered. For this purpose, analysis such as epigenetic analysis of RNA or acetylation other than methylation is necessary, and research on factors other than AO is necessary. Further investigation is necessary to ascertain the necessity of additional research wherein the optimal control group comprises individuals originating from the VHSMC.

In conclusion, we demonstrated that AO-exposure-related CpG sites play a role as epigenetic factors in type 2 diabetes pathogenesis. Comprehensive epigenetic profiling revealed that the effect of AO on diabetes pathogenesis was characterised by significant epigenetic changes in the various gene categories involved. Furthermore, while conducting a comparative study of the PRS data based on genotype, it was observed that there were no statistically significant disparities between individuals with AO-exposed and AO-unexposed type 2 diabetes. However, notable distinctions were identified when comparing these individuals to the control group consisting of normal individuals. This finding suggests that individuals who possess a genetic susceptibility to type 2 diabetes may have epigenetic alterations that are linked to the onset of type 2 diabetes. These findings suggest that additional research is required, including a causal investigation of the DMPs and additional putative experimental validation.

## Data availability statement

The authors declare that the data supporting the findings of this study are available within the article and its supplementary information is available in **Supplementary Figures 1**–**7** and **Supplementary Tables 1**–**8**. The data supporting the findings of this study for genetic and epigenetic data in Veterans Hospital are available upon reasonable request. However, access to Korea Biobank data for research for control is available upon reasonable request under the permission of the National Biobank of Korea, which can be contacted at http://nih.go.kr/biobank/cmm/main/mainPage.do?/ and via e-mail biobank@korea.kr.

## Ethics statement

The studies involving humans were approved by the institutional review board (IRB) of the Veterans Health Service Medical Center (VHSMC) (IRB no. 2018-12-016 and IRB no. 2018-08-032). For control data, the IRB of the VHSMC approved the study and waived informed consent from the individuals (IRB no. 2019-06-007) because data analysis was retrospective manner. The studies were conducted in accordance with the local legislation and institutional requirements. The participants provided their written informed consent to participate in this study.

## Author contributions

SS: Conceptualization, Data curation, Formal Analysis, Investigation, Methodology, Software, Visualization, Writing – original draft, Writing – review & editing. YAK: Conceptualization, Data curation, Resources, Supervision, Writing – original draft, Writing – review & editing. YL: Data curation, Methodology, Resources, Supervision, Writing – original draft, Writing – review & editing. YJK: Resources, Software, Supervision, Writing – original draft, Writing – review & editing. B-JK: Data curation, Formal Analysis, Supervision, Writing – original draft, Writing – review & editing. JA: Conceptualization, Data curation, Formal Analysis, Investigation, Methodology, Writing – original draft, Writing – review & editing. HJ: Data curation, Formal Analysis, Writing – original draft, Writing – review & editing. AD: Formal Analysis, Software, Writing – original draft, Writing – review & editing. KP: Formal Analysis, Methodology, Writing – original draft, Writing – review & editing. SW: Validation, Writing – original draft, Writing – review & editing, Conceptualization, Data curation, Investigation, Methodology, Project administration, Resources, Software, Supervision. JHS: Conceptualization, Data curation, Funding acquisition, Investigation, Methodology, Project administration, Resources, Supervision, Validation, Writing – original draft, Writing – review & editing.

## Supplementary material

Supplementary Data Sheet 2 Polygenetic risk analysis.

Supplementary Data Sheet 3 The EPIC-Norkfolk study.
