# Supplementary material for: Epigenetic link between Agent Orange exposure and type 2 diabetes in Korean veterans
Source: Front Endocrinol (Lausanne). 2024 Jul 12;15:1375459. doi: 10.3389/fendo.2024.1375459 (PMC11272593; doi:10.3389/fendo.2024.1375459)
Supplement: Supplementary file 1 [file Table_1.docx]

**Supplementary Table S1.** **Summary of estimated proportion of leukocyte using Houseman method**

| Mean (SD) | CD4 T cell | CD8T cell | Granulocyte | Monocyte | NK cell | Nucleated RBC | B cell |
| --- | --- | --- | --- | --- | --- | --- | --- |
| AO-exposed  T2D | 0.093 (0.04) | 0.105 (0.04) | 0.521 (0.11) | 0.095 (0.02) | 0.087 (0.05) | 0.044 (0.01) | 0.049 (0.03) |
| AO-unexposed T2D | 0.122 (0.04) | 0.133 (0.04) | 0.474 (0.09) | 0.088 (0.02) | 0.060 (0.04) | 0.047 (0.01) | 0.069 (0.03) |
| Healthy | 0.131 (0.04) | 0.145 (0.04) | 0.461 (0.09) | 0.084 (0.02) | 0.053 (0.04) | 0.046 (0.01) | 0.072 (0.03) |

AO, Agent Orange, T2D, type 2 diabetes

**Supplementary Table S2. log2(fold-change) and summary statistics of seven CpGs**

|  |  |  |  | | | | AO-exposed T2D  vs AO-unexposed T2D | | | | AO-exposed T2D vs Healthy | | | | AO-unexposed T2D vs Healthy | | | |
| --- | --- | --- | --- | --- | --- | --- | --- | --- | --- | --- | --- | --- | --- | --- | --- | --- | --- | --- |
| CpG | | | | Chr | Position | Gene | logFC | t | p-value | Adj.  p-value | logFC | t | p-value | Adj.  p-value | logFC | t | p-value | Adj.  p-value |
| cg07553761 | | | | 3 | 160167977 | *TRIM59* | -0.33 | -10.54 | 6.51E-25 | 8.91E-20 | -0.34 | -11.50 | 3.74E-29 | 1.54E-23 | -0.01 | -0.29 | 7.69E-01 | 9.86E-01 |
| cg20075319 | | | | 1 | 112332718 | *KCND3* | 0.26 | 7.85 | 8.92E-15 | 9.40E-11 | 0.30 | 9.79 | 7.61E-22 | 5.21E-17 | 0.04 | 1.93 | 5.45E-02 | 7.79E-01 |
| cg21757266 | | | | 13 | 28896815 | *FLT1* | 0.20 | 7.37 | 3.09E-13 | 1.83E-09 | 0.25 | 9.63 | 3.26E-21 | 1.91E-16 | 0.04 | 2.42 | 1.56E-02 | 6.72E-01 |
| cg05203217 | | | | 20 | 43730072 | *KCNS1* | 0.31 | 7.01 | 4.03E-12 | 1.62E-08 | 0.35 | 8.38 | 1.48E-16 | 2.64E-12 | 0.04 | 1.21 | 2.26E-01 | 8.99E-01 |
| cg20102280 | | | | 13 | 47470793 | *HTR2A* | 0.29 | 6.13 | 1.15E-09 | 1.78E-06 | 0.352 | 8.04 | 2.12E-15 | 2.69E-11 | 0.06 | 2.05 | 4.08E-02 | 7.56E-01 |
| cg26081717 | | | | 9 | 104249747 | *TMEM246* | 0.25 | 6.57 | 7.47E-11 | 1.87E-07 | 0.281 | 7.94 | 4.46E-15 | 4.88E-11 | 0.03 | 1.26 | 2.07E-01 | 8.92E-01 |
| cg21878650 | | | | 5 | 64558623 | *ADAMTS6* | 0.28 | 5.55 | 3.57E-08 | 2.76E-05 | 0.369 | 7.77 | 1.67E-14 | 1.46E-10 | 0.09 | 2.55 | 1.08E-02 | 6.40E-01 |

**Supplementary Table S3.** **Validation of the identified T2D associated DMPs in EPIC-Norfolk**

|  |  |  | **VHSMC + KoGES** | | **EPIC-Norfolk** | |
| --- | --- | --- | --- | --- | --- | --- |
| **CpG** | **Chr** | **Position** | **estimate** | ***p*-value** | **estimate** | ***p*-value** |
| cg19693031 | 1 | 145441552 | -0.258 | 1.32E-15 | -0.647 | 2.72E-21 |
| cg00574958 | 11 | 68607622 | -0.210 | 2.01E-12 | -0.371 | 5.21E-09 |
| cg17058475 | 11 | 68607737 | -0.214 | 3.70E-12 | -0.091 | 1.45E-01 |
| cg09737197 | 11 | 68607675 | -0.175 | 6.85E-11 | 0.006 | 9.21E-01 |
| cg26826927 | 7 | 107103667 | 0.109 | 4.23E-08 | 0.076 | 2.04E-01 |
| cg21878650 | 5 | 64558623 | 0.168 | 7.56E-08 | 0.053 | 4.04E-01 |
| cg02610360 | 11 | 120197521 | 0.145 | 1.60E-07 | 0.118 | 4.95E-02 |
| cg07553761 | 3 | 160167977 | -0.102 | 3.49E-07 | 0.061 | 3.89E-01 |
| cg20102280 | 13 | 47470793 | 0.147 | 3.52E-07 | 0.106 | 9.85E-02 |
| cg07504977 | 10 | 102131012 | 0.103 | 4.05E-07 | 0.242 | 1.88E-04 |
| cg23378546 | 17 | 76352717 | 0.184 | 8.36E-07 | 0.151 | 2.34E-02 |
| cg17462962 | 5 | 154347281 | 0.109 | 8.97E-07 | -0.057 | 3.37E-01 |
| cg16834320 | 3 | 195167302 | 0.115 | 2.66E-06 | 0.088 | 1.51E-01 |
| cg18625627 | 14 | 81426015 | 0.110 | 3.19E-06 | 0.124 | 8.50E-02 |
| cg11202345 | 17 | 76976057 | 0.140 | 3.25E-06 | 0.256 | 9.56E-05 |
| cg05203217 | 20 | 43730072 | 0.126 | 4.75E-06 | 0.057 | 3.44E-01 |
| cg05339515 | 16 | 68624615 | -0.177 | 5.96E-06 | -0.021 | 7.25E-01 |
| cg10619342 | 15 | 65369005 | 0.109 | 6.65E-06 | -0.011 | 8.64E-01 |

DMP, differentially methylated position; EPIC-Norfolk, European Prospective Investigation into Cancer and Nutrition-Norfolk Study; KoGES, Korean Genome and Epidemiology Study; T2D, type 2 diabetes; VHSMC, Veterans Health Service Medical Center

**Supplementary Table S4. Results of subgroups analyses for diabetes mellitus microvascular complications**

|  | **All** | | | **DM CKD** | | | **DM retinopathy** | | | |
| --- | --- | --- | --- | --- | --- | --- | --- | --- | --- | --- |
| **CpG** | **log_2_FC** | **t** | **p-value** | **log_2_FC** | **t** | ***p*-value** | **log_2_FC** | **t** | ***p*-value** |  |
| cg07553761 | -0.348 | -6.732 | 1.13E-10 | -0.338 | -6.055 | 4.59E-09 | -0.348 | -6.732 | 1.13E-10 |  |
| cg20075319 | 0.316 | 6.546 | 3.29E-10 | 0.198 | 3.128 | 1.95E-03 | 0.316 | 6.546 | 3.29E-10 |  |
| cg05203217 | 0.359 | 4.865 | 2.02E-06 | 0.348 | 3.857 | 1.43E-04 | 0.359 | 4.865 | 2.02E-06 |  |
| cg21878650 | 0.330 | 4.729 | 3.77E-06 | 0.283 | 3.300 | 1.10E-03 | 0.330 | 4.729 | 3.77E-06 |  |
| cg21757266 | 0.163 | 4.130 | 4.93E-05 | 0.166 | 3.358 | 8.94E-04 | 0.163 | 4.130 | 4.93E-05 |  |
| cg20102280 | 0.277 | 4.034 | 7.28E-05 | 0.259 | 2.926 | 3.72E-03 | 0.277 | 4.034 | 7.28E-05 |  |
| cg26081717 | 0.210 | 3.769 | 2.04E-04 | 0.222 | 3.131 | 1.93E-03 | 0.210 | 3.769 | 2.04E-04 |  |

CKD, chronic kidney disease; DM, diabetes mellitus; FC, fold change

**Supplementary Table S5. CpG-SNP pairs determined using methylation quantitative trait locus analysis**

| **Chr** | **CpG** | **Site** | **SNP** | **Distance from CpG** | **Estimate** | ***p*-value** | **Adjusted *p*-value** | **SNP-T2D**  **p-value** |
| --- | --- | --- | --- | --- | --- | --- | --- | --- |
| 1 | cg25215028 | 21759308 | rs4654947 | -45740 | -0.123 | 8.69E-14 | 1.31E-11 | 0.546 |
|  |  |  | rs10799701 | -61682 | -0.049 | 1.74E-03 | 3.58E-02 | 0.476 |
|  |  |  | rs1697421 | -63984 | 0.128 | 4.84E-19 | 1.13E-16 | 0.242 |
|  |  |  | rs7520712 | -71855 | -0.118 | 2.68E-15 | 4.92E-13 | 0.794 |
|  |  |  | rs869180 | -80422 | 0.094 | 2.55E-10 | 2.62E-08 | 0.948 |
|  | cg25334541 | 33908001 | rs10914640 | 446271 | 0.073 | 1.43E-04 | 4.34E-03 | 0.203 |
|  |  |  | rs111376301 | 415492 | -0.069 | 2.97E-04 | 8.29E-03 | 0.916 |
|  |  |  | rs12723104 | 147804 | 0.131 | 5.57E-11 | 6.22E-09 | 0.811 |
|  |  |  | rs11584564 | 55991 | 0.126 | 3.12E-14 | 5.01E-12 | 0.401 |
|  |  |  | rs4652879 | 23437 | 0.221 | 3.66E-29 | 1.57E-26 | 0.704 |
|  |  |  | rs1535920 | -2936 | 0.198 | 1.06E-08 | 9.06E-07 | 0.916 |
|  | cg17018193 | 41261492 | rs10889608 | 26315 | 0.048 | 5.91E-04 | 1.46E-02 | 0.465 |
|  |  |  | rs9970530 | -6822 | -0.129 | 1.15E-11 | 1.47E-09 | 0.898 |
|  |  |  | rs4660469 | -24861 | 0.067 | 1.66E-06 | 9.08E-05 | 0.076 |
|  |  |  | rs6697721 | -31509 | -0.050 | 1.20E-03 | 2.65E-02 | 0.537 |
|  |  |  | rs1041239 | -34050 | 0.074 | 1.13E-05 | 4.58E-04 | 0.165 |
|  |  |  | rs963377 | -35919 | 0.054 | 1.75E-04 | 5.16E-03 | 0.612 |
|  |  |  | rs61778974 | -41093 | 0.059 | 2.45E-03 | 4.80E-02 | 0.568 |
|  |  |  | rs7543690 | -184577 | -0.125 | 9.01E-07 | 5.39E-05 | 0.783 |
|  |  |  | rs12123384 | -443526 | -0.059 | 2.16E-03 | 4.36E-02 | 0.296 |
|  | cg19433225 | 78512997 | rs750720 | 91212 | -0.211 | 1.02E-04 | 3.19E-03 | 0.056 |
|  |  |  | rs515662 | -17621 | 0.317 | 4.94E-28 | 1.81E-25 | 0.061 |
|  |  |  | rs685393 | -66364 | -0.163 | 4.00E-08 | 2.94E-06 | 0.913 |
|  |  |  | rs7542998 | -77030 | -0.218 | 4.41E-07 | 2.76E-05 | 0.934 |
|  | cg20075319 | 112332718 | rs474860 | 117506 | -0.069 | 3.85E-06 | 1.77E-04 | 0.789 |
|  |  |  | rs552802 | 74173 | -0.077 | 1.24E-06 | 7.26E-05 | 0.703 |
|  |  |  | rs197374 | 42735 | -0.083 | 2.62E-08 | 2.10E-06 | 0.315 |
|  |  |  | rs1538390 | 5951 | -0.082 | 7.35E-04 | 1.73E-02 | 0.093 |
|  |  |  | rs11102358 | -155522 | -0.060 | 2.92E-04 | 8.24E-03 | 0.843 |
|  |  |  | rs12404392 | -168120 | -0.060 | 1.48E-03 | 3.16E-02 | 0.308 |
|  | cg26784032 | 215697002 | rs12028008 | 465456 | -0.065 | 1.88E-04 | 5.48E-03 | 0.070 |
|  |  |  | rs2363561 | 441695 | -0.071 | 3.66E-05 | 1.25E-03 | 0.063 |
|  | cg01421737 | 224647109 | rs17575128 | -285461 | -0.129 | 7.83E-05 | 2.55E-03 | 0.157 |
| 2 | cg22400605 | 162975451 | rs72870422 | 19395 | 0.071 | 2.52E-06 | 1.25E-04 | 0.544 |
|  |  |  | rs16846294 | 11577 | 0.113 | 3.97E-08 | 2.94E-06 | 0.412 |
|  |  |  | rs10165543 | -13630 | -0.181 | 2.45E-36 | 1.57E-33 | 0.979 |
| 3 | cg07578119 | 39332018 | rs11917223 | 8595 | 0.057 | 9.99E-04 | 2.27E-02 | 0.985 |
|  |  |  | rs6783639 | 7953 | 0.069 | 6.00E-05 | 2.03E-03 | 0.788 |
|  |  |  | rs1317871 | -148500 | 0.119 | 1.60E-03 | 3.37E-02 | 0.564 |
|  |  |  | rs2233204 | -222768 | 0.106 | 2.22E-05 | 8.39E-04 | 0.157 |
|  | cg05778738 | 107308392 | rs12489299 | 322712 | -0.052 | 2.48E-03 | 4.82E-02 | 0.099 |
|  | cg16834320 | 195167302 | rs2279630 | 297960 | -0.108 | 3.58E-08 | 2.78E-06 | 0.353 |
|  |  |  | rs59190596 | 266163 | 0.117 | 1.64E-03 | 3.42E-02 | 0.317 |
|  |  |  | rs9825185 | 232668 | -0.181 | 1.36E-12 | 1.94E-10 | 0.602 |
|  |  |  | rs113429880 | 165295 | 0.173 | 1.89E-06 | 9.72E-05 | 0.149 |
|  |  |  | rs7622180 | 163669 | -0.142 | 1.47E-15 | 2.90E-13 | 0.719 |
|  |  |  | rs7634345 | 93899 | -0.162 | 9.42E-11 | 1.01E-08 | 0.156 |
|  |  |  | rs9837749 | -3564 | -0.111 | 3.98E-07 | 2.56E-05 | 0.339 |
| 5 | cg25735583 | 95681194 | rs4336380 | 66334 | -0.054 | 9.95E-04 | 2.27E-02 | 0.063 |
|  |  |  | rs12186664 | 50969 | -0.091 | 2.28E-07 | 1.54E-05 | 0.138 |
|  |  |  | rs156022 | -62514 | 0.063 | 1.15E-04 | 3.55E-03 | 0.877 |
|  |  |  | rs3762986 | -89668 | -0.052 | 1.25E-03 | 2.72E-02 | 0.956 |
|  | cg00811436 | 142962690 | rs1373999 | 68307 | 0.071 | 1.88E-06 | 9.72E-05 | 0.682 |
|  |  |  | rs1838714 | 44858 | -0.066 | 1.65E-06 | 9.08E-05 | 0.635 |
|  |  |  | rs152223 | 4976 | -0.108 | 1.44E-14 | 2.47E-12 | 0.661 |
|  |  |  | rs153516 | -16493 | 0.068 | 6.23E-07 | 3.81E-05 | 0.649 |
|  |  |  | rs17100500 | -41039 | 0.067 | 1.87E-06 | 9.72E-05 | 0.474 |
|  | cg17462962 | 154347281 | rs73290716 | 348180 | -0.101 | 4.88E-04 | 1.24E-02 | 0.715 |
|  |  |  | rs255529 | 130980 | -0.208 | 2.13E-21 | 5.46E-19 | 0.320 |
|  |  |  | rs10515728 | 17266 | 0.166 | 6.18E-32 | 3.17E-29 | 0.611 |
|  |  |  | rs13165424 | -5731 | -0.078 | 2.94E-05 | 1.03E-03 | 0.097 |
|  |  |  | rs6893162 | -56596 | -0.137 | 2.09E-06 | 1.05E-04 | 0.295 |
|  |  |  | rs351290 | -99475 | -0.192 | 6.03E-43 | 7.75E-40 | 0.844 |
|  |  |  | rs76578229 | -134981 | 0.126 | 2.83E-05 | 1.02E-03 | 0.728 |
|  |  |  | rs17117134 | -265807 | 0.080 | 3.88E-04 | 1.03E-02 | 0.263 |
|  | cg10392263 | 177036887 | rs34582406 | 16855 | -0.154 | 5.85E-22 | 1.67E-19 | 0.292 |
|  |  |  | rs6600950 | 3368 | -0.051 | 4.41E-04 | 1.13E-02 | 0.564 |
| 6 | cg06827192 | 154730156 | rs2499649 | 54241 | 0.079 | 1.47E-04 | 4.39E-03 | 0.767 |
|  |  |  | rs6907452 | 31596 | 0.084 | 1.44E-05 | 5.62E-04 | 0.540 |
|  |  |  | rs3734275 | 7430 | -0.115 | 4.02E-09 | 3.69E-07 | 0.185 |
|  |  |  | rs9478538 | -9600 | -0.098 | 3.02E-04 | 8.36E-03 | 0.819 |
|  |  |  | rs11155973 | -20349 | 0.102 | 2.13E-07 | 1.48E-05 | 0.765 |
|  |  |  | rs9397718 | -23313 | -0.080 | 1.20E-04 | 3.68E-03 | 0.545 |
|  |  |  | rs6910758 | -30819 | -0.122 | 3.26E-04 | 8.73E-03 | 0.784 |
|  |  |  | rs201488977 | -32718 | -0.080 | 3.98E-04 | 1.04E-02 | 0.511 |
| 7 | cg26826927 | 107103667 | rs6466145 | 484655 | 0.046 | 6.23E-04 | 1.52E-02 | 0.052 |
|  |  |  | rs6466154 | 448222 | -0.080 | 4.31E-09 | 3.81E-07 | 0.088 |
|  |  |  | rs56182569 | 447909 | 0.052 | 1.90E-04 | 5.48E-03 | 0.052 |
|  |  |  | rs2707378 | 366460 | 0.074 | 7.42E-04 | 1.73E-02 | 0.257 |
|  |  |  | rs113285340 | 361158 | 0.083 | 1.00E-05 | 4.15E-04 | 0.633 |
|  |  |  | rs11760261 | 324187 | 0.039 | 2.58E-03 | 4.95E-02 | 0.295 |
|  |  |  | rs74457173 | 319433 | 0.104 | 2.49E-05 | 9.13E-04 | 0.434 |
|  |  |  | rs6971774 | 269528 | 0.121 | 4.15E-11 | 5.02E-09 | 0.099 |
|  |  |  | rs6949634 | 118983 | -0.150 | 5.93E-25 | 1.90E-22 | 0.065 |
|  |  |  | rs6466165 | 102932 | -0.069 | 7.21E-08 | 5.15E-06 | 0.161 |
|  |  |  | rs4730243 | 36081 | -0.062 | 2.89E-06 | 1.37E-04 | 0.312 |
|  |  |  | rs10953542 | -146032 | 0.060 | 6.46E-05 | 2.15E-03 | 0.412 |
|  |  |  | rs17405606 | -167696 | -0.107 | 1.14E-05 | 4.58E-04 | 0.052 |
| 8 | cg03830443 | 106333948 | rs285823 | 231768 | 0.081 | 8.20E-04 | 1.90E-02 | 0.838 |
| 9 | cg16504526 | 73025362 | rs10868841 | -93302 | 0.043 | 5.38E-04 | 1.34E-02 | 0.438 |
|  | cg26081717 | 104249747 | rs1929494 | 12973 | 0.085 | 9.20E-06 | 3.94E-04 | 0.915 |
|  |  |  | rs67224495 | 7969 | -0.120 | 8.84E-06 | 3.85E-04 | 0.586 |
|  |  |  | rs9299345 | -90065 | -0.071 | 1.26E-03 | 2.72E-02 | 0.577 |
|  |  |  | rs10989573 | -149297 | -0.066 | 7.26E-04 | 1.73E-02 | 0.915 |
|  |  |  | rs1016429 | -152617 | -0.133 | 1.38E-05 | 5.45E-04 | 0.987 |
| 10 | cg07504977 | 102131012 | rs11190480 | 18937 | 0.085 | 2.63E-06 | 1.28E-04 | 0.054 |
|  |  |  | rs3793767 | 15266 | -0.040 | 1.69E-03 | 3.49E-02 | #N/A |
|  |  |  | rs10883465 | 8825 | 0.086 | 1.32E-09 | 1.26E-07 | 0.063 |
|  |  |  | rs653341 | -13220 | 0.064 | 2.05E-03 | 4.18E-02 | 0.095 |
|  |  |  | rs6584366 | -22444 | 0.060 | 1.85E-05 | 7.08E-04 | 0.093 |
| 11 | cg09737197 | 68607675 | rs7112615 | 77357 | -0.114 | 1.59E-03 | 3.37E-02 | 0.215 |
|  |  |  | rs3794021 | 39850 | -0.116 | 2.91E-04 | 8.24E-03 | 0.978 |
|  |  |  | rs2924689 | 24004 | -0.141 | 7.30E-04 | 1.73E-02 | 0.590 |
|  | cg07845298 | 77181561 | rs4945168 | 175407 | -0.085 | 2.26E-05 | 8.43E-04 | 0.237 |
|  |  |  | rs531638 | 34314 | 0.093 | 3.23E-06 | 1.51E-04 | 0.454 |
|  |  |  | rs3133456 | -19177 | -0.135 | 4.30E-11 | 5.02E-09 | 0.078 |
|  |  |  | rs12222461 | -21210 | 0.073 | 1.26E-03 | 2.72E-02 | 0.133 |
|  | cg02610360 | 120197521 | rs7934236 | 4010 | 0.111 | 6.43E-04 | 1.56E-02 | 0.364 |
|  |  |  | rs3809059 | -8642 | 0.055 | 1.07E-03 | 2.41E-02 | 0.179 |
|  | cg09975274 | 128017166 | rs12049869 | 298938 | -0.054 | 3.99E-04 | 1.04E-02 | 0.555 |
|  |  |  | rs11221110 | 217719 | -0.086 | 1.33E-08 | 1.10E-06 | 0.638 |
|  |  |  | rs7107087 | 154424 | -0.067 | 9.11E-05 | 2.89E-03 | 0.804 |
|  |  |  | rs10750388 | 51395 | 0.158 | 1.97E-17 | 4.22E-15 | 0.062 |
|  |  |  | rs55897421 | 48109 | -0.198 | 2.83E-41 | 2.42E-38 | 0.853 |
|  |  |  | rs948206 | -6292 | -0.353 | 3.81E-131 | 9.78E-128 | 0.072 |
|  |  |  | rs9787850 | -59430 | -0.055 | 5.17E-04 | 1.30E-02 | 0.308 |
|  |  |  | rs7112731 | -98574 | -0.117 | 4.00E-10 | 3.95E-08 | 0.383 |
|  |  |  | rs565536 | -119223 | 0.048 | 2.58E-03 | 4.95E-02 | 0.510 |
|  |  |  | rs10893844 | -168684 | -0.096 | 2.83E-07 | 1.86E-05 | 0.064 |
| 13 | cg20102280 | 47470793 | rs73175542 | -8072 | -0.153 | 6.81E-05 | 2.24E-03 | 0.692 |
|  | cg25984701 | 52006860 | rs6561622 | 199672 | -0.066 | 2.39E-03 | 4.75E-02 | 0.786 |
|  |  |  | rs9316546 | -51862 | -0.157 | 2.85E-05 | 1.02E-03 | 0.846 |
| 14 | cg18625627 | 81426015 | rs8022788 | 136579 | -0.056 | 2.43E-03 | 4.80E-02 | 0.153 |
|  |  |  | rs7144208 | 12526 | -0.089 | 1.47E-06 | 8.39E-05 | 0.262 |
|  |  |  | rs2300518 | -32501 | 0.056 | 1.15E-03 | 2.57E-02 | 0.685 |
|  |  |  | rs58417382 | -42431 | -0.072 | 8.10E-05 | 2.60E-03 | 0.500 |
| 16 | cg05339515 | 68624615 | rs2274239 | -101168 | 0.115 | 5.94E-06 | 2.68E-04 | 0.360 |
|  |  |  | rs9989407 | -139897 | 0.118 | 3.61E-05 | 1.25E-03 | 0.323 |
| 17 |  |  | rs4789908 | 13998 | -0.080 | 3.26E-04 | 8.73E-03 | 0.789 |
|  |  |  | rs9891726 | -16044 | -0.089 | 6.48E-06 | 2.87E-04 | 0.364 |
|  |  |  | rs80269200 | -20451 | 0.143 | 1.70E-12 | 2.30E-10 | 0.236 |
| 19 | cg13882251 | 40772266 | rs11880261 | -16375 | 0.057 | 3.11E-04 | 8.49E-03 | 0.420 |
|  |  |  | rs74768924 | -65411 | 0.143 | 9.77E-06 | 4.12E-04 | 0.533 |
| 21 | cg11525951 | 43641170 | rs220318 | 121064 | 0.057 | 2.23E-03 | 4.47E-02 | 0.814 |

**p*-values at a significance level of 0.05, adjusted using the Benjamini–Hochberg method

Chr, chromosome; SNP, single nucleotide polymorphism

**Supplementary Table S6. Results of Reverse Mendelian randomisation analysis**

| Chr | CpG Site | Mapinfo | Estimate | SE | *p*-value |
| --- | --- | --- | --- | --- | --- |
| 1 | cg25215028 | 21759308 | 1.4611 | 1.1368 | 0.1990 |
| 1 | cg09142571 | 32222876 | -0.7892 | 1.0603 | 0.4569 |
| 1 | cg25334541 | 33908001 | -0.0101 | 0.8999 | 0.9910 |
| 1 | cg17018193 | 41261492 | 1.2581 | 1.0336 | 0.2238 |
| 1 | cg19433225 | 78512997 | 0.5186 | 1.5745 | 0.7420 |
| 1 | cg20075319 | 112332718 | 0.0956 | 0.7521 | 0.8989 |
| 1 | cg05028010 | 145437567 | -0.2379 | 1.0755 | 0.8250 |
| 1 | cg02988288 | 145440445 | -2.3966 | 1.9697 | 0.2240 |
| 1 | cg26974062 | 145440734 | -2.4337 | 1.9290 | 0.2073 |
| 1 | cg19693031 | 145441552 | -2.6001 | 1.9375 | 0.1799 |
| 1 | cg26784032 | 215697002 | 1.6780 | 1.2911 | 0.1940 |
| 1 | cg01421737 | 224647109 | -0.0086 | 0.9155 | 0.9925 |
| 2 | cg21217117 | 65190910 | -0.0989 | 1.2034 | 0.9345 |
| 2 | cg03173169 | 75060921 | 1.4616 | 1.1642 | 0.2096 |
| 2 | cg13585080 | 145780395 | -0.5713 | 0.8987 | 0.5251 |
| 2 | cg22400605 | 162975451 | 0.0604 | 0.7575 | 0.9365 |
| 3 | cg07578119 | 39332018 | 1.3881 | 1.1399 | 0.2236 |
| 3 | cg05778738 | 107308392 | -0.1787 | 0.8515 | 0.8338 |
| 3 | cg07553761 | 160167977 | -0.9746 | 0.7547 | 0.1968 |
| 3 | cg06603936 | 169383031 | -1.0175 | 1.3874 | 0.4635 |
| 3 | cg16834320 | 195167302 | 1.3057 | 1.1868 | 0.2715 |
| 4 | cg01156663 | 154360462 | 1.2918 | 1.3697 | 0.3458 |
| 5 | cg21878650 | 64558623 | 2.3420 | 1.8447 | 0.2045 |
| 5 | cg25735583 | 95681194 | 1.4239 | 1.2520 | 0.2556 |
| 5 | cg00811436 | 142962690 | 0.3446 | 0.7954 | 0.6649 |
| 5 | cg17462962 | 154347281 | 1.6493 | 1.2576 | 0.1900 |
| 5 | cg10392263 | 177036887 | 1.0873 | 0.9991 | 0.2767 |
| 6 | cg00857282 | 16130727 | 1.0934 | 1.7094 | 0.5226 |
| 6 | cg06827192 | 154730156 | 2.5041 | 1.7925 | 0.1627 |
| 7 | cg26826927 | 107103667 | 0.7250 | 0.7993 | 0.3646 |
| 7 | cg08189223 | 144107509 | 1.9263 | 1.4189 | 0.1749 |
| 8 | cg03830443 | 106333948 | 1.8155 | 1.5127 | 0.2303 |
| 8 | cg07023764 | 130567184 | 0.9669 | 0.8857 | 0.2752 |
| 9 | cg16504526 | 73025362 | 0.6124 | 0.7539 | 0.4168 |
| 9 | cg26081717 | 104249747 | 1.8322 | 1.4287 | 0.1999 |
| 10 | cg21634264 | 21065467 | -0.0416 | 1.5887 | 0.9791 |
| 10 | cg07504977 | 102131012 | 1.7518 | 1.3059 | 0.1801 |
| 11 | cg00574958 | 68607622 | -1.0376 | 1.3070 | 0.4274 |
| 11 | cg09737197 | 68607675 | -0.4703 | 1.3220 | 0.7221 |
| 11 | cg05325763 | 68607719 | -1.0133 | 1.3512 | 0.4535 |
| 11 | cg17058475 | 68607737 | -1.8677 | 1.8073 | 0.3016 |
| 11 | cg07845298 | 77181561 | 1.2444 | 1.2483 | 0.3190 |
| 11 | cg02610360 | 120197521 | -0.4434 | 0.9431 | 0.6383 |
| 11 | cg09975274 | 128017166 | -0.1568 | 0.8455 | 0.8529 |
| 12 | cg19699676 | 111142773 | 1.0714 | 1.1617 | 0.3566 |
| 13 | cg21757266 | 28896815 | 0.7807 | 0.7402 | 0.2918 |
| 13 | cg26544530 | 28896826 | 2.0655 | 1.4132 | 0.1442 |
| 13 | cg20102280 | 47470793 | 1.4445 | 1.2356 | 0.2426 |
| 13 | cg25984701 | 52006860 | 0.2252 | 1.1602 | 0.8461 |
| 14 | cg18625627 | 81426015 | -0.7759 | 1.0478 | 0.4592 |
| 15 | cg03685481 | 51066872 | 0.3422 | 0.8389 | 0.6835 |
| 15 | cg10619342 | 65369005 | 0.8099 | 0.9852 | 0.4112 |
| 15 | cg07024568 | 71615957 | 0.5917 | 0.9731 | 0.5432 |
| 16 | cg05339515 | 68624615 | -0.4430 | 1.3442 | 0.7418 |
| 17 | cg23378546 | 76352717 | 1.3359 | 1.4744 | 0.3651 |
| 17 | cg11202345 | 76976057 | 1.5724 | 1.4147 | 0.2666 |
| 19 | cg13882251 | 40772266 | 0.4334 | 0.8371 | 0.6048 |
| 20 | cg05203217 | 43730072 | -0.0183 | 1.0014 | 0.9854 |
| 21 | cg11525951 | 43641170 | 1.3225 | 1.2409 | 0.2868 |

Chr, chromosome; SE, standard error

| Phenotype | Set | PRS.R^2^ | p-value | Num_SNP | Competitive.P |
| --- | --- | --- | --- | --- | --- |
| T2D | KEGG_ADIPOCYTOKINE_SIGNALING_PATHWAY | 9.22E-04 | 3.70E-01 | 140 | 0.602 |
| AO | KEGG_ADIPOCYTOKINE_SIGNALING_PATHWAY | 7.48E-04 | 5.10E-01 | 140 | 0.761 |
| T2D | KEGG_APOPTOSIS | 1.17E-04 | 7.50E-01 | 143 | 0.939 |
| AO | KEGG_APOPTOSIS | 6.00E-06 | 9.53E-01 | 143 | 0.998 |
| T2D | KEGG_INSULIN_SIGNALING_PATHWAY | 2.06E-03 | 1.81E-01 | 241 | 0.337 |
| AO | KEGG_INSULIN_SIGNALING_PATHWAY | 5.12E-04 | 5.86E-01 | 241 | 0.825 |
| T2D | KEGG_MATURITY_ONSET_DIABETES_OF_THE_YOUNG | 1.92E-03 | 1.97E-01 | 37 | 0.353 |
| AO | KEGG_MATURITY_ONSET_DIABETES_OF_THE_YOUNG | 8.96E-04 | 4.71E-01 | 37 | 0.718 |

**Supplementary Table S7**. **Summary of enrichment test of pathway-specific PRS**

AO, Agent Orange, T2D, type 2 diabetes, PRS.R^2^, Variance explained by the PRS, Num_SNP, Number of SNPs included in the pathway, Competitive.P, competitive p-value

**Supplementary Table S8**. **Sargan test result for six significant CpGs in T2D MR analysis**

| CpG | Statistics | Degree of freedom | p-value |
| --- | --- | --- | --- |
| cg22400605 | 1.509 | 1 | 0.219 |
| cg26826927 | 13.472 | 11 | 0.264 |
| cg06827192 | 6.206 | 6 | 0.401 |
| cg20075319 | 3.161 | 4 | 0.531 |
| cg26081717 | 0.018 | 3 | 0.999 |
| cg20102280 | *Not available* | | |

T2D, type 2 diabetes, All meQTLs for each CpGs are used as instrumental variables for Sargan test. cg20102280 has only one meQLT, so Saragn test is not available.
