# Supplementary material for: Epigenetic link between Agent Orange exposure and type 2 diabetes in Korean veterans
Source: Front Endocrinol (Lausanne). 2024 Jul 12;15:1375459. doi: 10.3389/fendo.2024.1375459 (PMC11272593; doi:10.3389/fendo.2024.1375459)
Supplement: Supplementary Data Sheet 2 — Polygenetic risk analysis. [file DataSheet_1.docx]

**Supplementary Figure S1.** **Detailed workflow and the number of individuals and markers**

**
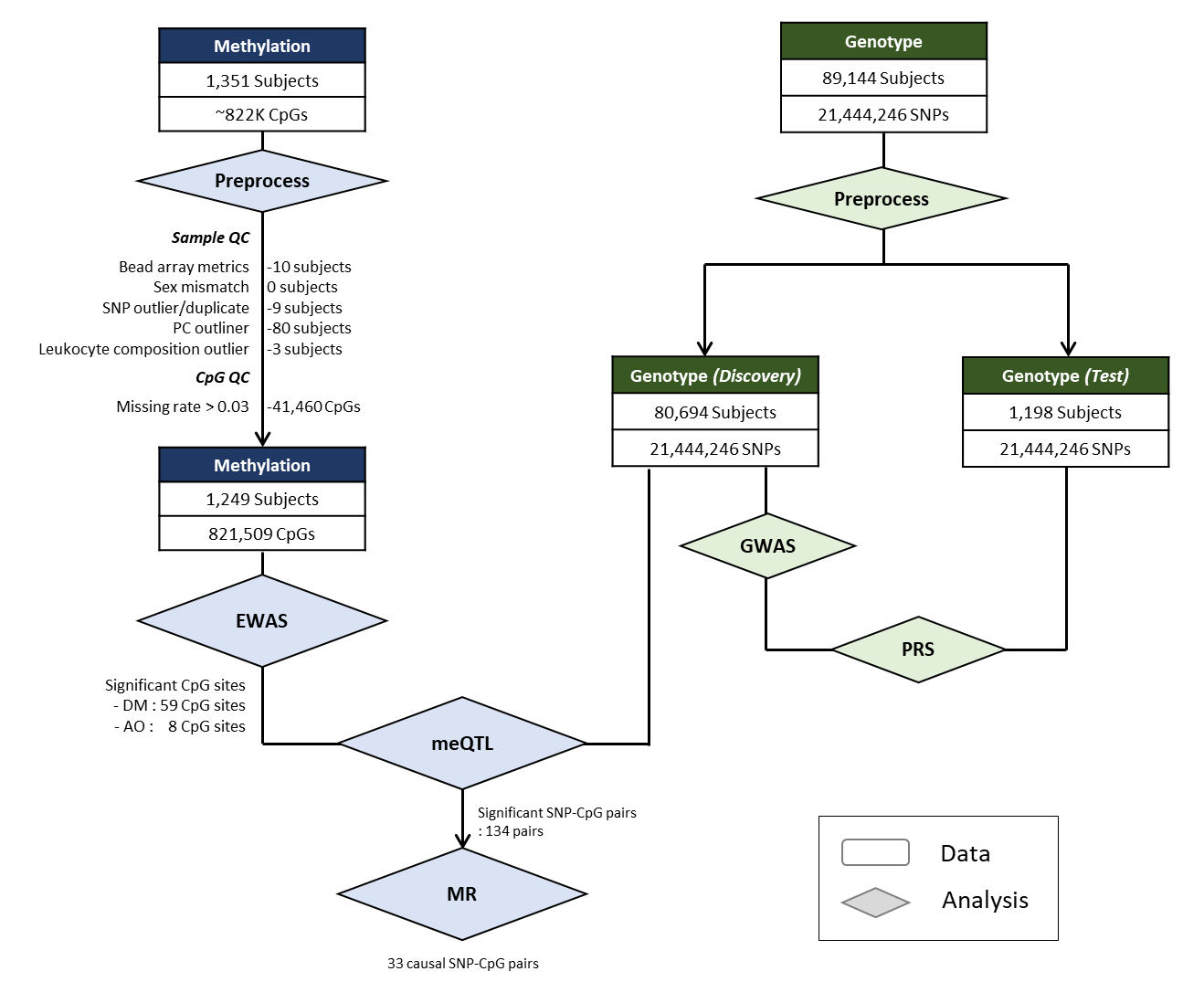
**

**Supplementary Figure S2. MDS plot and Q-Q plots** : (a) MDS plot after QC and batch effect correction; (b) Q-Q plot for the T2D versus healthy individuals; (c) Q-Q plot for the AO-exposed T2D individuals versus AO-unexposed T2D individuals; (d) the change in the $\log_{2} fold change$ and *p*-values of 52 significant CpG sites in the T2D versus the healthy group, but not in AO-exposed T2D versus AO-unexposed T2D.


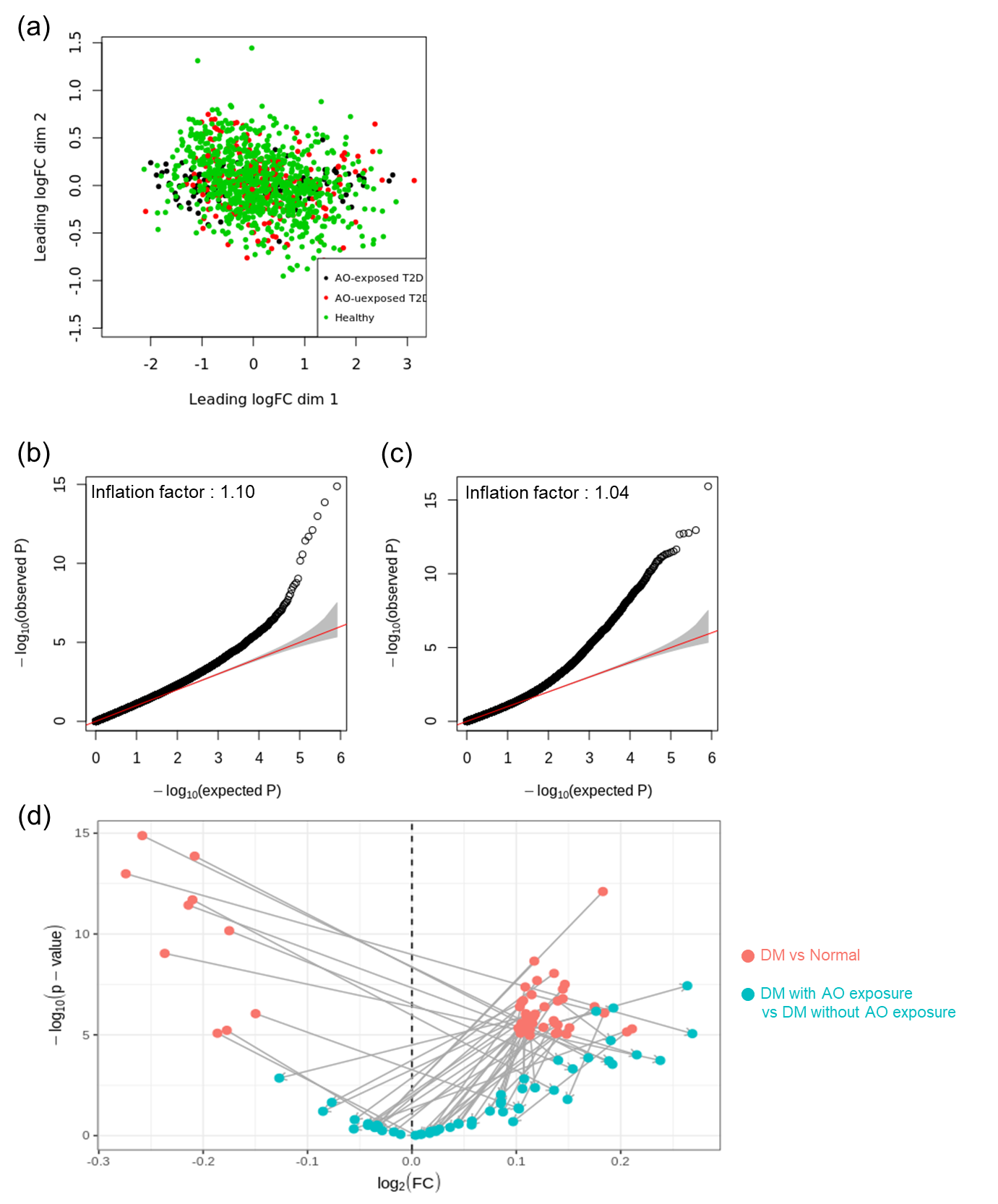


T2D, Type 2 diabetes; AO, Agent Orange; FC, fold-change; PCA, principal component analysis

**Supplementary Figure S3. Sensitivity analysis according to elderly group, male, and non-cancer history patient of 7 CpG, identified in primary analysis**


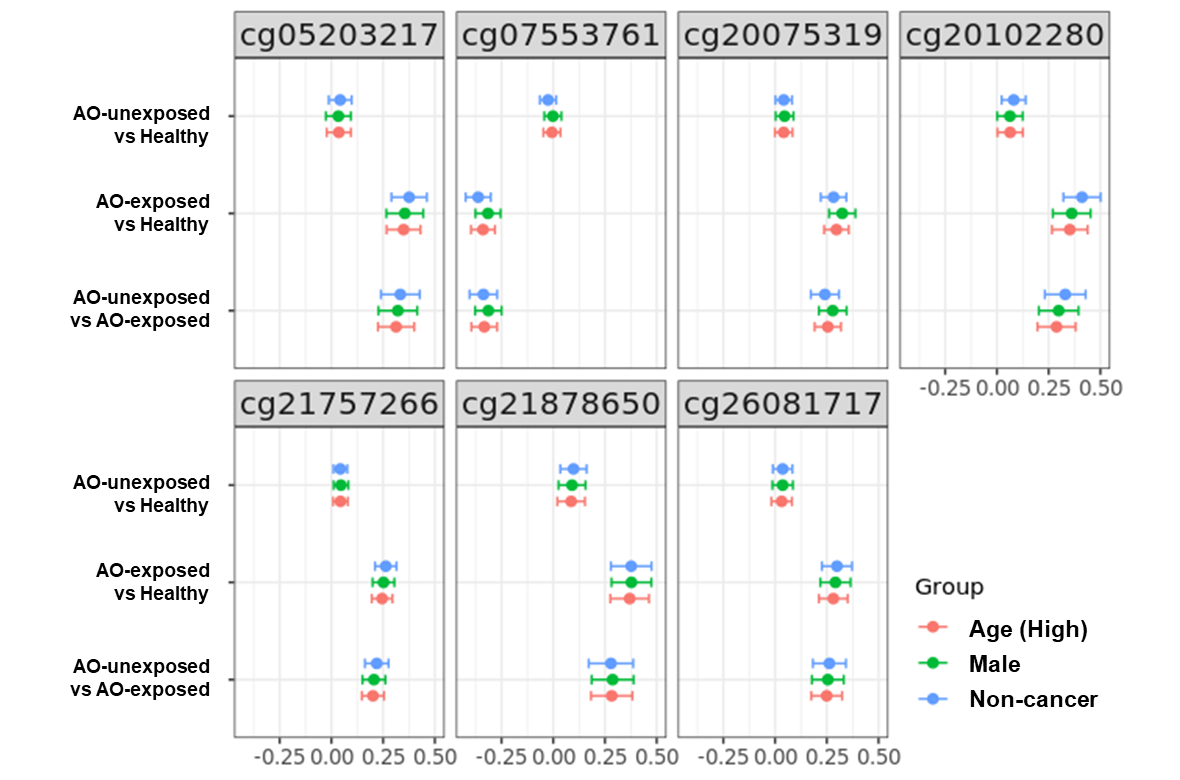


The log_2_FC values of seven identified CpGs in the sensitivity analysis, when considering only the elder group, male group or subjectss without a history of cancer, consistently demonstrate the same direction and effect size.

AO, Agent Orange; FC, fold change

**Supplementary Figure S4. Bar graph showing the number of single nucleotide polymorphism pairs for each CpG site.**


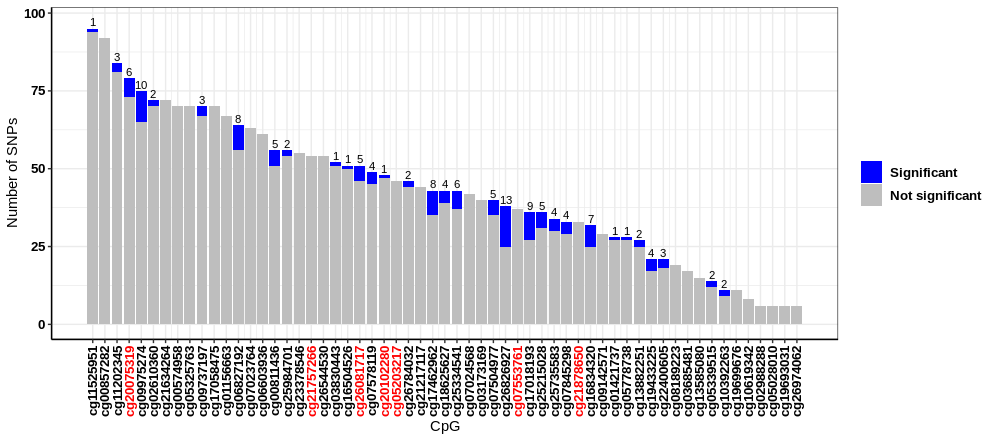


One hundred and thirty-four significant SNP-CpG pairs are highlighted in blue and the CpGs significant for Agent Orange (AO) are highlighted in red.

SNP, single nucleotide polymorphism

**Supplementary Figure S5**. **Significant cis-CpG –SNPs determined using** **methylation quantitative trait locus analysis**


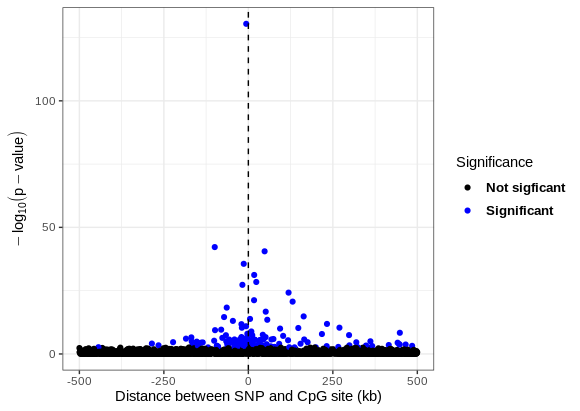


*cis-*SNPs within 500 kb from the CpGs that had a significant association with type 2 diabetes were considered significant using test correlations for 2,569 SNP-CpG pairs in the 59 CpG sites.

SNP, single nucleotide polymorphism

**Supplementary Figure S6.** **Comparison of the polygenic risk score between AO-exposed individuals with type 2 diabetes**


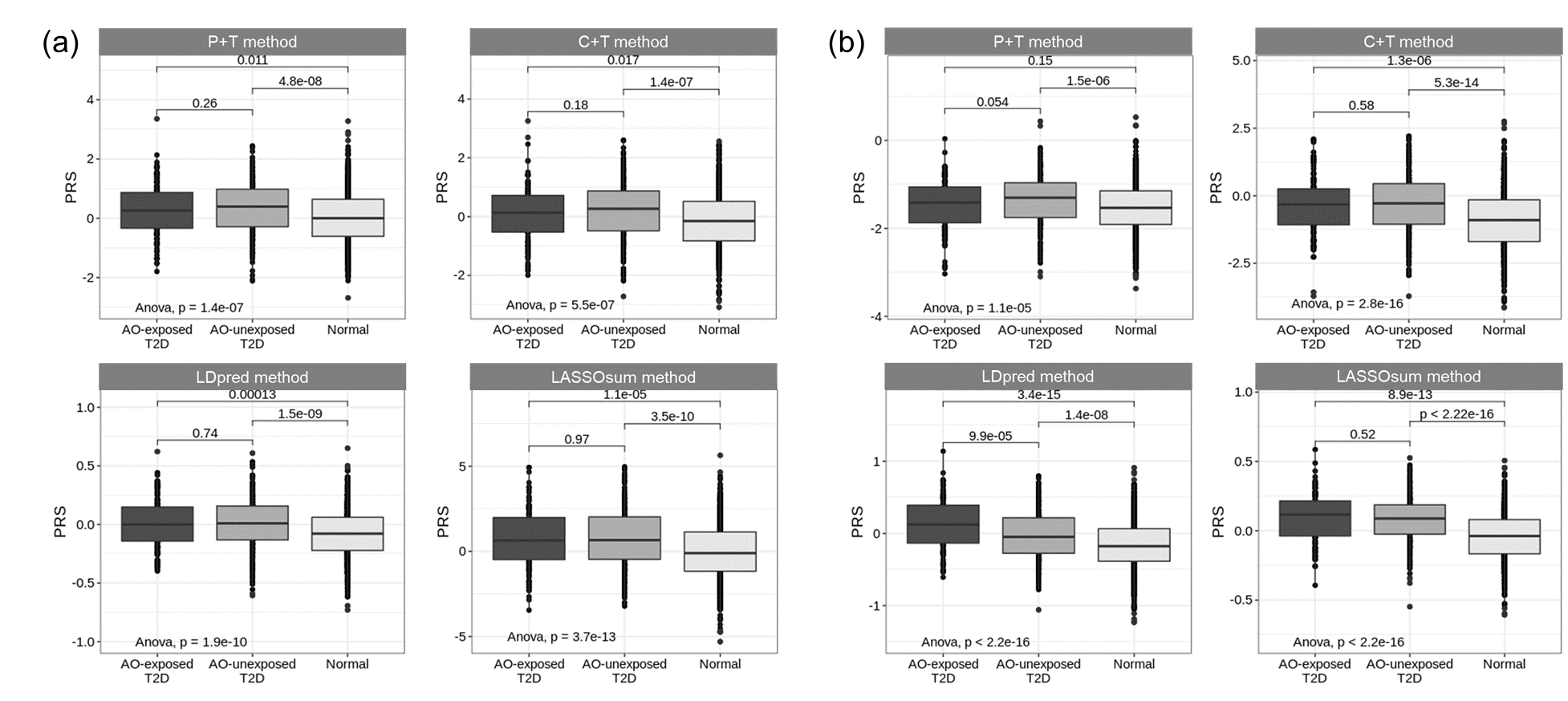


(a) Reference data adopted from the Korean-Northeast Asian Reference Database (NARD) imputed dataset. Both AO-exposed and AO-unexposed groups of individuals with type 2 diabetes had higher polygenic risk score (PRS) values than healthy individuals, and there was no significant difference between the AO-exposed and AO-unexposed type 2 diabetes groups in all PRS models (*p* > 0.05).

(b) Reference data adopted from Biobank Japan (BBJ) summary statistics. Both AO-exposed type 2 diabetes and AO-unexposed type 2 diabetes individuals had higher PRS values than normal controls, and there was no significant difference between the AO-exposed type 2 diabetes and AO-unexposed type 2 diabetes groups according to the C + T and LASSOsum methods (*p* > 0.05). There was a difference among the three groups according to the LDpred approach, but there was also a difference according to other methods; thus, the interpretation was limited.

AO, Agent Orange; T2D, type 2 diabetes; P+T method, Pruning and thresholding; C+T method, clumping and thresholding; PRS, polygenic risk score


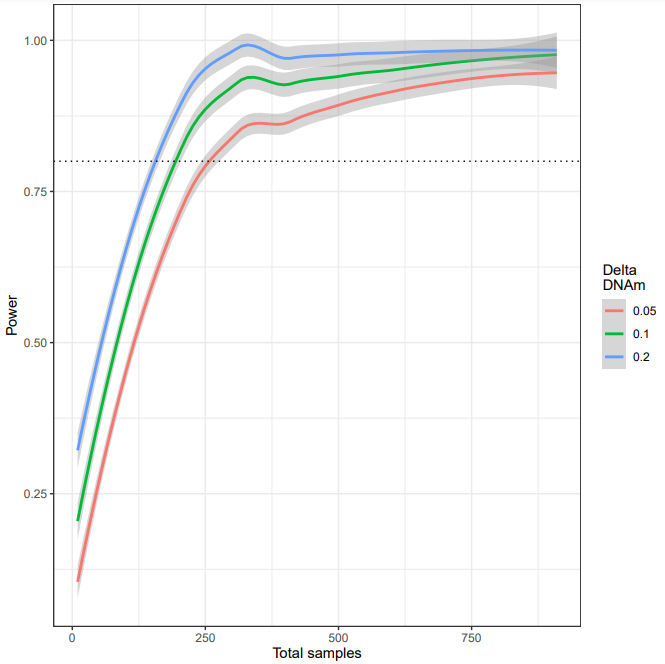
**Supplementary Figure S7.** **Power calculation to assess the adequacy of sample size**

Power calculation for EWASs over increasing total sample sizes (assuming the two comparison groups to have equal sizes), and for various differences in DNAm levels. Plots were generated using pwrEWAS. The simulation accounted for tissue type (blood samples), number of total and differentially methylated CpGs (80,000, 50), effect size (0.05, 0.1, 0.2), target false discovery rate (0.05) , and statistical methods to perform differential methylation analyses(limma).
