# Supplementary material for: Epigenetic link between Agent Orange exposure and type 2 diabetes in Korean veterans
Source: Front Endocrinol (Lausanne). 2024 Jul 12;15:1375459. doi: 10.3389/fendo.2024.1375459 (PMC11272593; doi:10.3389/fendo.2024.1375459)
Supplement: Supplementary Data Sheet 3 — The EPIC-Norkfolk study. [file DataSheet_2.docx]

**Supplemental Methods and results for estimation of Polygenic Risk Scores for Type 2 Diabetes Development**

**Results**

The PRS of individuals of both type 2 diabetes groups (AO-exposed and AO-unexposed individuals with type 2 diabetes) was higher than that of healthy individuals (*p* < 0.05, **Additional file 1: Fig. S5**). However, no significant differences in PRS values were found between the AO-exposed individuals with type 2 diabetes and the AO-unexposed individuals (*p* > 0.05), except for the PRS determined using the LDpred method based on BBJ summary statistics (**Additional file 1: Fig. S5**). According to this method, less than 3% of the SNPs in the test dataset were matched to Hapmap3, which may explain this difference. These results from the group comparisons indicate that AO exposure has no genetic influence on the propensity of individuals to develop type 2 diabetes. These findings imply that the individuals with a genetic predisposition to type 2 diabetes may undergo epigenetic modifications under AO exposure, which are related to the development of type 2 diabetes.

Moreover, we conducted pathway-specific PRS calculations to probe the potential association between AO exposure and specific pathogenic pathways implicated in T2D. Pathways such as adipocytokine signaling pathway, apoptosis, insulin signalling and maturity onset diabetes of the young were delineated as distinct T2D-related pathways from KEGG. Through pathway enrichment comparisons across AO-unexposed versus AO-exposed groups, none exhibited statistically significant differences (**Additional file 2: Table. S7**).

**Method**

A genome-wide association study (GWAS) of 80,694 individuals from KoGES cohort was performed (**Figure** **3**). Logistic regression was conducted after adjusting for sex and age, and 10 PC scores were estimated from the genetic relationship matrix. Next, summary statistics were used to calculate the PRS. For replication analysis, summary statistics from GWAS and BioBank Japan (BBJ) were used (<https://pheweb.jp/>) [1]. Using these two summary statistics, the PRSs were calculated separately using Pruning and Thresholding, Clumping and Thresholding, the LDpred method, and the Lassosum method [2, 3]. For P + T and C + T, the significance threshold levels for pruning and clumping were set at *p* = 0.5 and *p* = 10^-5^, respectively. The SNPs obtained using HapMap3 were used in the LDpred method, and the LD structures of the Asian version hg19 (build 37) were used as reference panels for the Lassosum method.

Pathway specific PRS analyses were conducted using PRSice-2 v2.3.5[4]. Gene sets representing different pathways associated with T2D pathway were acquired from KEGG[5] via MSigDB[6, 7]. These sets were further annotated using GTF files obtained from ENSEMBL(GRCh37.75)[8]. For the calculation of pathway-specific PRS, SNPs within the gene regions corresponding to the genes within each pathway were included. To assess the enrichment of pathway specific PRS between AO groups, 10,000 permutations were executed, providing empirical competitive p-values that adjust for pathway size.

**Reference**

1. Sakaue, S., et al., *A cross-population atlas of genetic associations for 220 human phenotypes.* Nat Genet, 2021. **53**(10): p. 1415-1424.

2. Vilhjalmsson, B.J., et al., *Modeling Linkage Disequilibrium Increases Accuracy of Polygenic Risk Scores.* Am J Hum Genet, 2015. **97**(4): p. 576-92.

3. Mak, T.S.H., et al., *Polygenic scores via penalized regression on summary statistics.* Genet Epidemiol, 2017. **41**(6): p. 469-480.

4. Choi, S.W., et al., *PRSet: Pathway-based polygenic risk score analyses and software.* PLoS Genet, 2023. **19**(2): p. e1010624.

5. Kanehisa, M. and S. Goto, *KEGG: kyoto encyclopedia of genes and genomes.* Nucleic Acids Res, 2000. **28**(1): p. 27-30.

6. Subramanian, A., et al., *Gene set enrichment analysis: a knowledge-based approach for interpreting genome-wide expression profiles.* Proc Natl Acad Sci U S A, 2005. **102**(43): p. 15545-50.

7. Liberzon, A., et al., *Molecular signatures database (MSigDB) 3.0.* Bioinformatics, 2011. **27**(12): p. 1739-40.

8. Yates, A.D., et al., *Ensembl Genomes 2022: an expanding genome resource for non-vertebrates.* Nucleic Acids Res, 2022. **50**(D1): p. D996-D1003.
