# Supplementary material for: Epigenetic link between Agent Orange exposure and type 2 diabetes in Korean veterans
Source: Front Endocrinol (Lausanne). 2024 Jul 12;15:1375459. doi: 10.3389/fendo.2024.1375459 (PMC11272593; doi:10.3389/fendo.2024.1375459)
Supplement: Supplementary file 4 [file DataSheet_3.docx]

**Supplemental descriptions about the EPIC-Norfolk**

EPIC-Norfolk research is a prospective cohort initiated between 1993 and 1997 with 25,639 participants aged 40 to 79 years. This cohort, mirroring the general demographics of England and Wales, displayed notable characteristics such as 99.7% European heritage.

A sub-cohort was randomly selected from the EPIC-Norfolk study, excluding individuals with pre-existing diabetes at the study's onset. Various sources were utilized to identify incident T2D cases, including health questionnaires, self-reported information, drugs brought to clinical examinations, and linkage with medical records, incorporating external sources like general practice and hospital registers.

Individuals claiming a diabetes history without supporting evidence were not considered verified cases. The follow-up period ended at T2D, or mortality, whichever came first. Adhering to case-cohort design principles, the study encompassed both case participants from the random sub-cohort and others. However, for analysis, only the event case set was considered, with the comparison group comprising non-case patients. Baseline measures of BMI and HbA1c were collected for each participant.

The data used for EWAS was undertaken in an incident T2DM case-cohort study nested within the EPIC-Norfolk study. Methylation intensities were measured using Illumina HumanMethylation450 array in whole blood samples of 1,264 individuals, comprising of 563 T2D cases and 701 controls. Logistic regression was employed to determine the impact of DMPs on T2D after adjusting the effect of age, sex, estimated cell counts, and sample plate. Summary statistics derived from the logistic regression was downloaded from <https://www.repository.cam.ac.uk/handle/1810/299058>.
